# Oxidative Stress-Driven Mechanisms and Biomarkers of Drug-Induced Nephrotoxicity: Translational Insights and Therapeutic Implications

**DOI:** 10.3390/antiox15040412

**Published:** 2026-03-25

**Authors:** Rizwan Ahamad, Nida Mubin, Mohammed Alnukhali, Mohd Akhtar, Mohd Aqil, Mohd Mujeeb, Anis Ahmad

**Affiliations:** 1 Department of Pharmacology, School of Pharmaceutical Education and Research, Jamia Hamdard, New Delhi 110062, India; rahamad995@gmail.com (R.A.);; 2 Division of Hematology & Oncology, Case Western Reserve University, Cleveland, OH 44106, USA; nxm793@case.edu; 3 Department of Radiation Oncology, University of Miami Miller School of Medicine, Sylvester Comprehensive Cancer Center, Miami, FL 33136, USA; mxa1931@miami.edu; 4 Department of Biochemistry and Molecular Biology, University of Miami Miller School of Medicine, Miami, FL 33136, USA; 5 Department of Pharmaceutics, School of Pharmaceutical Education and Research, Jamia Hamdard, New Delhi 110062, India; 6 Department of Pharmacognosy and Phytochemistry, School of Pharmaceutical Education and Research, Jamia Hamdard, New Delhi 110062, India

**Keywords:** oxidative stress, drug-induced nephrotoxicity, reactive oxygen species (ROS), acute kidney injury, chronic kidney disease, renal tubular injury, redox signaling, mitochondrial dysfunction, kidney injury biomarkers, antioxidant therapy

## Abstract

Drug-induced kidney injury remains a major clinical challenge associated with diverse therapeutic agents and is an important cause of acute kidney injury, chronic renal dysfunction, and treatment-related morbidity. Growing evidence indicates that nephrotoxicity caused by anticancer, immunosuppressive, and anti-infective drugs is strongly driven by oxidative stress and redox homeostasis disruption. Excessive production of reactive oxygen species (ROS) in renal tubular cells overwhelms endogenous antioxidant defenses and triggers mitochondrial dysfunction, inflammatory signaling, and activation of stress-responsive pathways that culminate in tubular injury and renal functional decline. These processes promote apoptosis, necrosis, microvascular injury, and a reduction in the glomerular filtration rate, while dysregulation of redox-sensitive pathways involved in cell survival and repair further heightens renal vulnerability. This review summarizes current mechanistic insights into oxidative stress-mediated pathways of drug-induced nephrotoxicity, with emphasis on their translational relevance. In addition, it discusses emerging biomarkers for early detection and highlights recent advances in antioxidant-based and redox-modulating strategies that may help prevent renal injury and preserve kidney function.

## 1. Introduction

Renal involvement in drug toxicity is a serious clinical problem. It is one of the principal reasons for acute renal failure (ARF) in drug-treated patients, especially in those on anti-cancer, anti-bacterial, and anti-immune therapies [1]. Due to its immense metabolic activity, a rich complement of mitochondria, and its predominant role in xenobiotic metabolism, the kidney, and in particular the proximal tubule cells, is particularly susceptible to injury [2]. Even slight alterations in the renal cellular milieu can often be reflected in substantial impairment in renal function [3].

Accumulating evidence has shown that oxidative stress and redox imbalance play key pathogenic roles in drug-induced renal injury, but not as indirect consequences following cellular injury [4]. Common nephrotoxic medications, such as platinum-based chemotherapeutic agents, aminoglycoside antibiotics, calcineurin inhibitors, and alkylating drugs, cause overproduction of reactive oxygen species (ROS) in renal tubular cells [5]. When ROS production exceeds the buffering capacity of endogenous antioxidant systems, redox-sensitive signaling pathways become dysregulated, initiating maladaptive inflammatory and apoptotic responses [6]. Oxidative stress damage may result from dysfunctional mitochondria producing excessive ROS, since ROS are byproducts of adenosine triphosphate (ATP) synthesis [7].

At the cellular and molecular levels, oxidative stress is associated with mitochondrial integrity, lipid peroxidation, lipid membrane damage, and oxidative damage to DNA [8]. All these converge on stress-activated kinases and transcription factors, such as the mitogen-activated protein kinases and the p53 pathway, which contribute to the amplification of injury and death in the tubular cells [9]. Concurrently, the production of pro-inflammatory cytokines, such as tumor necrosis factor-alpha, catalyzed by ROS, further aggravates kidney injury [10]. The combined effects of cellular damage to tubular epithelial cells, impaired microvascular function, and chronic inflammation further contribute to reduced glomerular filtration rates and the subsequent progression to chronic renal disease [11].

Although impairment of the renal antioxidant defense system plays a significant role in drug-induced nephrotoxicity, multiple other mechanisms, including direct cytotoxicity, hemodynamic alterations, crystal deposition, and immune-mediated injury, also contribute to renal damage [12]. Under normal circumstances, renal cells maintain their normal functioning through redox signaling [10]. Disruption of these antioxidant defenses sensitizes the kidney to otherwise tolerable pharmacologic exposures, highlighting redox homeostasis as a critical determinant of renal resilience.

Given the ever-increasing reliance on potentially nephrotoxic therapies in oncology, transplantation, and infectious disease management, there is a compelling need for a more detailed elucidation of the mechanisms underlying oxidative stress-dependent renal injury [13,14]. Such information is necessary for the development of predictive biomarkers, Reno protective strategies, and antioxidant-based interventions that limit kidney damage without sacrificing therapeutic efficacy. This review synthesizes current mechanistic understanding of the role of oxidative stress-driven pathways in drug-induced nephrotoxicity, with particular emphasis on shared molecular nodes for disparate classes of drugs and their implications in antioxidant-targeted prevention and treatment.

## 2. Methodology

This narrative review has been carried out through the application of a structured, reproducible literature search process, which has enabled us to selectively identify pertinent literature on drug-induced nephrotoxicity, particularly focusing on oxidative stress, redox signaling, and antioxidant mechanisms [15]. This methodology has been applied to synthesize concepts, not for meta-analysis.

### 2.1. Literature Search Strategy

Literature searches were performed across several scientific databases, including PubMed/MEDLINE, Scopus, Web of Science, ScienceDirect, SciFinder, Springer, and Google Scholar, to capture primarily English-language peer-reviewed articles. In constructing the search queries, combinations of terms related to nephrotoxicity and oxidative stress-focused keywords were applied. Core search terms included combinations of:

- Nephrotoxicity, acute kidney injury, drug-induced kidney injury
- Oxidative stress, reactive oxygen species, redox imbalance, mitochondrial dysfunction
- Antioxidants, redox signaling, MAPK, p53, inflammation

Additional targeted searches were conducted for specific nephrotoxic agents discussed in this review, including cisplatin, cyclophosphamide, tacrolimus, and gentamicin, to ensure mechanistic depth across pharmacologic classes.

### 2.2. Study Selection and Inclusion Criteria

Articles were selected based on their relevance to renal injury mechanisms mediated by oxidative stress. Priority was given to:

- Experimental studies (in vitro and in vivo) elucidating ROS-dependent renal injury pathways.
- Clinical and translational studies linking oxidative stress biomarkers to kidney dysfunction.
- Review articles providing mechanistic frameworks relevant to redox biology in kidney disease.

Studies addressing exclusively non-renal toxicity, unrelated organ systems, or lacking mechanistic relevance to oxidative or redox processes were excluded. When cumulative evidence existed, more recent and highly cited studies, particularly those published after 2020, were prioritized.

### 2.3. Data Extraction and Thematic Organization

Information extracted from selected studies included:

- Source and type of nephrotoxic agent
- Renal cell types affected (with emphasis on proximal tubular epithelium)
- Mechanisms of ROS generation and redox imbalance
- Activation of stress-responsive signaling pathways (e.g., MAPK, p53)
- Links between oxidative stress, inflammation, and cell death
- Reported effects of antioxidant or redox-modulating interventions

Rather than summarizing studies individually, findings were integrated thematically to identify convergent molecular mechanisms shared across distinct nephrotoxic insults.

### 2.4. Focus on Oxidative Stress and Antioxidant Pathways

Consistent with the scope of *Antioxidants*, particular attention was given to studies examining:

- Mitochondrial sources of ROS in renal injury
- Dysregulation of endogenous antioxidant systems
- Redox-sensitive inflammatory and apoptotic signaling
- Biomarkers reflect oxidative damage in kidney disease

Where available, evidence supporting antioxidant-based strategies for mitigating nephrotoxicity was highlighted to provide a translational context.

## 3. Factors Causing Drug-Induced Nephrotoxicity

The renal clearance of drugs involves several carefully regulated processes, including glomerular filtration, tubular secretion, and tubular reabsorption [16]. During glomerular filtration, the non-protein-bound fraction of the drug, along with waste products, passes from the blood into the filtration. At the same time, the majority of bound substances remain in the blood. The filtrate, passing through the proximal tubules, leads to the reabsorption of some vital solutes, and further xenobiotics are actively secreted in the tubular lumen [17]. These processes, important for renal protection, impart remarkable susceptibility to renal cells by exposing them to relatively higher concentrations of toxic compounds and their metabolites [18].

Nephrotoxicity from drugs can affect the glomerulus, tubular epithelium, interstitial tissue, and blood vessels of the renal vasculature. The pathophysiology of renal dysfunction caused by these injuries can result from various factors, including changes in renal hemodynamics, direct tubular toxicity, inflammation, crystal deposition, rhabdomyolysis, and thrombotic microangiopathy [19].

### 3.1. Renal Hemodynamic

Renal function requires careful intraglomerular hemodynamic regulation to maintain normal glomerular filtration rate regardless of systemic blood pressure [20]. The normal individual maintains the glomerular filtration rate (GFR) through autoregulatory changes in the tone of the afferent and efferent arterioles that ensure intraglomerular capillary pressures and ensure sufficient filtration, respectively [21,22].

Prostaglandins and the renin-angiotensin system have complementary effects in the mechanism. Renal prostaglandins cause vasodilation of the afferent arteriole, especially in low-volume states, thereby increasing renal blood flow [23]. Angiotensin II, on the other hand, primarily causes vasoconstriction of the efferent arteriole, thereby maintaining intraglomerular pressure and GFR in low-flow situations [24].

Pharmacological inhibition of these mechanisms can cause nephrotoxicity due to altered hemodynamics [25]. Prostaglandins can be inhibited by nonsteroidal anti-inflammatory drugs (NSAIDs), whose mechanism of action affects the efferent arteriole, leading to reduced renal flow [26]. Similarly, angiotensin-converting enzyme inhibitors (ACEIs) and angiotensin receptor blockers (ARBs) attenuate angiotensin II-mediated efferent arteriolar constriction, resulting in decreased intraglomerular pressure [27]. Despite the long-term protective effects of ACE inhibitors and ARBs on renal function in diabetic nephropathy, their administration in conditions of relative volume depletion, hypotension, and reduced renal perfusion can further decrease the already reduced GFR and cause acute kidney injury [28].

Impaired renal hemodynamics not only led to reduced filtration but also established a background of secondary tubular injury [29]. Perfusion and oxygenation defects accelerate renal hypoxia, which, in turn, exacerbates mitochondrial dysfunction and stimulates the production of free radicals [30]. The hemodynamic-oxidative vicious cycle intensifies the susceptibility of renal tubules, establishing a chain of injury from pre-renal underperfusion to drug nephrotoxicity [31]. Once its autoregulatory capacity is exceeded, failure of glomerular pressure and renal perfusion becomes a universal pre-renal injury mechanism in drug nephrotoxicity [32].

### 3.2. Renal Tubular Toxicity

Renal tubular toxicity is considered to be among the most common causes of drug-induced nephrotoxicity [33]. Within the renal tubule, the proximal tubule is particularly sensitive due to its high metabolic activity, high mitochondrial content, and its role in the uptake, secretion, and reabsorption of drugs [34]. The result is exposure of proximal tubular cells to higher concentrations of nephrotoxic substances and their metabolites [35].

A few drugs, such as the aminoglycosides and amphotericin B antibiotics, antiviral medications such as adefovir, and chemotherapy medications such as cisplatin, directly damage tubular cells [36,37]. There is evidence from animal studies suggesting that injury to the tubules is closely associated with mitochondrial toxicity, disruption of oxidative phosphorylation, and overproduction of ROS [38].

Damage to mitochondrial membranes by ROS and disruption of the cell’s calcium balance render cells non-viable [39]. Oxidative stress further compromises tubular transport systems by altering membrane lipids and transport proteins, thereby impairing resorptive and secretory capacity [40]. Sustained redox imbalance activates [20] stress-responsive signaling pathways and promotes apoptotic and necrotic cell death. As tubular epithelial integrity deteriorates, epithelial permeability increases, allowing filtrate to leak into the interstitial compartment. This back-leakage reduces effective tubular flow and contributes to a decline in GFR [39].

The susceptibility of PT cells can be further increased by their high expression level of cytochrome P-450 and various transporters, which facilitate the entry and metabolism of drugs [39]. Indeed, in some instances, certain biotransformation reactions in tubular cells produce reactive intermediates that can further augment oxidative damage [41]. On the other hand, certain toxic compounds can alter intercellular junctions and the cytoskeleton, leading to increased tubular resistance and elevated luminal pressure, thereby exacerbating functional damage [31].

Taken together, mitochondrial damage, oxidative stress, and transporter dysregulation provide a unifying framework for understanding cell death and renal impairment mediated by tubular cell injury [42]. Such phenomena delineate the pivotal role of redox imbalance in tubular cell toxicity and also mark the proximal tubules as a key target for antioxidant therapy to limit nephrotoxicity associated with medications [25].

### 3.3. Nephron Inflammation

Inflammation is a major pathological mechanism of drug-induced nephrotoxicity and often plays a role in the transition between cellular damage and chronic renal dysfunction [43]. Renal drugs can induce an inflammatory response in various renal tissues, including glomeruli, tubular epithelial cells, and the interstitial matrix. Chronic inflammation contributes to the accumulation of extracellular matrix and renal fibrosis, leading to nephron inactivity [44].

Inflammatory damage is usually an accompanying response to direct damage to tubulin or the endothelium. Drug-induced oxidation becomes a crucial mechanism in this damage because it activates transcription factors and cytokine signaling pathways involved in the process [45]. Overproduction of ROS triggers the release of pro-inflammatory mediators, such as tumor necrosis factor-alpha, interleukins, and chemokines, in kidney tissues during the inflammatory response [39].

Acute interstitial nephritis is a recognized inflammatory response to drug-related kidney injuries and is mostly seen with non-steroidal medicines and antibiotics like rifampicin. This form of kidney injury is seen with immune cell infiltration, interstitial edema, and tubular dysfunction [46]. On the contrary, chronic interstitial nephritis is a consequence of prolonged nephrotoxic exposure from medicines such as calcineurin inhibitors, lithium, analgesics, and several cancer medicines. This provokes a persistent inflammatory process, leading to fibroblast activation, collagen deposition, and, over time, kidney fibrosis [47].

Glomerular inflammation contributes to nephrotoxicity, especially in cases of immune-mediated drug reactions. Glomerulonephritis can occur concomitantly with proteinuria due to damage to the glomerular filtration barrier [48]. Oxidative injury contributes to glomerular damage by harming glomerular endothelial cells and podocytes, which, in turn, exacerbate glomerular inflammation and filtration defects [49].

Importantly, inflammation and oxidative stress are interlinked events, rather than distinct pathological processes. Indeed, ROS are both inducers and products of the inflammatory process, thereby leading to a vicious cycle of oxidative and inflammatory events [50]. The imbalance between inflammation and redox processes can thus accelerate chronic nephron degeneration and delayed recovery. Anti-inflammatory pathways, along with antioxidant mechanisms, can therefore be potential strategies to limit nephrotoxic effects of drugs and prevent the transition to chronic kidney disease [51].

### 3.4. Crystal Nephropathy

Crystal nephropathy represents one type of drug-induced kidney damage with intrarenal precipitation of poorly soluble drugs or their metabolites, causing tubular obstruction and resultant inflammation. It presents with progressive decline in renal function when crystal deposition alters tubular flow and function [31].

Certain drugs, such as sulfonamides, ciprofloxacin, ampicillin, and triamterene, are less soluble in urine and tend to become supersaturated when drug concentrations are high or when urine becomes more alkaline or acidic [52]. Supersaturation of these drugs enhances crystal formation in the renal tubules, especially in the distal nephron segments. Deposited crystals can obstruct tubules and alter filtrate flow by increasing intratubular pressure [53].

In addition to architectural obstruction, crystal injury initiates an inflammatory response and contributes to oxidative stress injury in tubular epithelial cells [54]. Irritation from obstruction and epithelial injury stimulates oxidative stress pathways, leading to an influx of reactive oxygen metabolites that further disrupt epithelial cell membranes [55]. This dual process of obstruction and oxidative stress injuries potently exacerbates tubular dysfunction with consequent nephron loss.

Crystal nephropathy is often seen in patients with pre-existing renal disease, dehydration, and reduced urine output, all of whom facilitate intratubular drug accumulation [56]. In the context of oncology, tumor lysis syndrome is an important example, where there is massive cellular turnover due to chemotherapy, resulting in an overproduction of uric acid. Uric acid crystallization in renal tubules causes acute tubular necrosis, resulting in renal failure [57].

In summary, the lessons from crystal nephropathy are clear: solubility, infusion regimens, and water intake are critical factors in preventing nephrotoxicity. The contribution of oxidative stress to tubular injury from crystal formation further supports the potential utility of redox therapy in preventing renal injury from intrarenal crystal formation.

### 3.5. Rhabdomyolysis

Rhabdomyolysis is a serious systemic process characterized by the massive breakdown of skeletal muscle and the subsequent release of its cellular contents into the bloodstream [58]. Myoglobin and Creatine Kinase are the main cellular components involved in the pathogenesis of renal damage in the spillage associated with rhabdomyolysis, as their levels become pathological due to their abundance in muscles [59].

Various etiologies have contributed to rhabdomyolysis, including trauma, immobilization, ischemic events, hyperthermia, metabolic disorders, substance abuse, and drug reactions [60]. Pharmacologic drugs such as statins, sympathomimetics, anticholinergic drugs, antihistamines, and some street drugs have contributed to cases of drug-induced rhabdomyolysis, both alone or with predisposing factors of dehydration or electrolyte disturbances [41].

Renal damage in the case of rhabdomyolysis results mainly from tubular damage caused by the cytotoxic effect of myoglobin. The kidneys selectively clear myoglobin, which accumulates in tubular cells, where it causes luminal and cellular damage [61]. Perhaps most significant, however, is the fact that myoglobin is a potent oxidant. Its structurally integral porphyrin ring catalyzes the conversion of oxygen into reactive oxygen species, which cause lipid peroxidation, mitochondrial damage, and DNA damage in tubular cells [41].

In addition to oxidative injury, myoglobin-induced vasoconstriction and endothelial damage further impair renal perfusion, contributing to the ischemic injury [62]. The combination of tubular injury, oxidative injury, and microvascular damage leads to acute tubular necrosis and a decline in glomerular filtration rate. Starting an intervention promptly could prevent the progression to acute renal failure due to rhabdomyolysis-related nephrotoxicity [63].

Rhabdomyolysis indicates a common pathway in which systemic injury and drug exposure result in oxidative stress-dependent renal injury mechanisms [64]. This underscores the importance of early recognition, aggressive hydration, and therapeutic strategies to limit oxidative injury and prevent permanent renal damage [65].

### 3.6. Thrombotic Microangiopathy (TMA)

Thrombotic microangiopathy (TMA) is a severe and potentially fatal syndrome of drug-induced nephrotoxicity due to microvascular endothelial damage, plate thrombi, and occlusive thrombosis in the renal arterioles and capillaries [66]. This process causes decreased blood flow and reduced filtration, triggering a sudden loss of renal function [67].

There are several pharmacologic agents associated with the development of drug-induced TMA, including immunosuppressants such as cyclosporine and tacrolimus, chemotherapy agents such as mitomycin-C, and others such as quinine [68]. These medications can directly damage renal endothelial cells or trigger an immune response, leading to endothelial dysfunction [49]. This alters endothelial hemostasis, leading to platelet activation and aggregation, as well as endothelial injury [69].

Oxidative stress has been shown to play a key role in the development of TMA. Overproduction of reactive oxygen species accompanies drug-induced endothelial damage. Additionally, oxidative stress reduces nitric oxide availability, a key factor in vasoconstriction and thrombosis. This imbalance makes the vessel intima even more fragile. Gradually, thrombi begin to accumulate within the glomerular and peritubular capillaries.

Additionally, microangiopathic injury results from mechanical trauma to peripheral erythrocytes passing through partially occluded vessels, thereby contributing to hemolysis and exacerbating tissue hypoxia [70]. Chronic microvascular obstruction and ischemia then culminate in glomerular sclerosis and tubular dysfunction. Left unattended, drug-associated TMA can result in permanent renal failure [67].

Overall, thrombotic microangiopathy constitutes a crossroads of endothelial toxicity, oxidative stress, and microvascular dysfunction. Indeed, recognizing redox-mediated endothelial injury as a central component of TMA suggests the potential for therapeutic interventions to prevent drug-induced renal failure by preserving endothelial integrity and mitigating oxidative damage.

## 4. The Mechanisms of Renal Injuries Caused by Medicinal Agents and Their Metabolites

Drug metabolism and elimination are multifunctional processes that involve not only the liver but also other organs, including the gastrointestinal tract and kidneys [71]. Medicinal agents and their metabolites are cleared via renal or extrarenal pathways. In the kidney, the major mechanisms of drug elimination are glomerular filtration and tubular secretion, processes that inherently expose renal cells, especially tubular epithelial and interstitial cells, to high concentrations of potentially injurious compounds [17].

During renal excretion, filtered drugs are transported from the glomerulus and come into contact with the apical membranes of tubular cells. Concomitantly, there is secretion of various compounds across the basolateral membranes of the proximal tubular cells, and these substances are secreted from the tubular lumen [72]. These tubular functions and mechanisms make the renal tubule highly susceptible to injury. As certain drugs and their metabolites move through the nephron from the renal tubule to the loop of Henle and then to the distal portions, their concentrations, solubility, and luminal pH change, leading to cast formation and tubular obstruction [73]. Apart from direct toxicity and obstruction, there could be induction of tubulointerstitial injury, which results in interstitial nephritis and renal dysfunction [74,75].

Mechanistically, the renal toxicity of drugs can be generally categorized into three closely intertwined processes [33]. First, direct tubular damage in the form of acute tubular necrosis occurs in a dose-dependent manner due to cellular accumulation of drugs or their reactive metabolites after apical uptake or basolateral secretion. Typically, these processes are associated with mitochondrial dysfunction and excessive reactive oxygen species production, both of which disrupt cellular energy homeostasis and promote cell death in tubular epithelial cells [76]. Second, tubular obstruction occurs due to the formation of drug or its metabolite crystals within the intraluminal space, thereby restricting filtrate flow and raising intratubular pressure. Finally, tubulo-interstitial inflammation occurs either as a consequence of cellular damage or immune reactions, in both of which the final common effector is the resultant oxidative injury, which exerts a crucial synergistic effect on inflammation and fibrosis [77].

These mechanisms rarely occur in isolation but rather interact to drive functional decline of the kidney. A comprehensive overview of pharmacologic agents and associated patterns of renal injury is shown in Table 1 below.

In clinical settings, nephrotoxicity frequently occurs during combination drug therapy, such as chemotherapeutic agents administered with antibiotics or immunosuppressants, and may manifest as additive or synergistic oxidative injury. Accumulating evidence indicates that synergistic nephrotoxicity arises when co-administered drugs converge on shared renal targets, including proximal tubular uptake, mitochondrial dysfunction, and excessive reactive oxygen species (ROS) generation, thereby overwhelming endogenous antioxidant defenses such as glutathione and antioxidant enzymes [36,85,86]. At the molecular level, multiple nephrotoxic agents activate common redox-sensitive signaling pathways, including MAPK, p53, and NF-κB, leading to enhanced inflammatory signaling and tubular cell death [9,45,46]. These mechanistic intersections provide a biological rationale for the heightened renal injury observed with combinations such as cisplatin and aminoglycosides or calcineurin inhibitors and underscore the importance of dose optimization, avoidance of high-risk drug combinations, and intensified renal monitoring in patients receiving combination therapies [87,88].

In addition, nephrotoxicity often develops in the context of concurrent injury to other organs, including the liver, heart, or lungs. Injury-induced systemic responses—such as the release of acute-phase proteins, inflammatory cytokines, oxidative stress mediators, and endothelial dysfunction can lower the renal injury threshold and amplify tubular inflammation through inter-organ crosstalk mechanisms [45,49]. These interactions highlight the importance of integrated organ monitoring and avoidance of overlapping toxicities when managing drugs associated with multi-organ adverse effects [86,87].

### 4.1. Anticancer Drugs

#### 4.1.1. Cisplatin

Cisplatin is a widely used platinum-based chemotherapeutic agent, although its clinical potential is limited by significant nephrotoxicity. Early clinical research studies had reported renal impairment as a major adverse reaction of cisplatin therapy; since then, numerous studies have observed that nearly one-third of patients subjected to cisplatin therapy develop objectively assessed renal impairment [84,89]. Typically observed clinically is cisplatin-induced nephrotoxicity, which occurs 7–10 days after therapy onset and is manifested by increased serum creatinine levels, reduced glomerular filtration rate, and electrolyte disturbances, such as hypomagnesemia and hypokalemia [85]. Despite an unclear characterization of the sequelae of long-term renal exposure to cisplatin, there is increasing evidence that even subclinical renal injury can induce a stable reduction in renal functional capacity [90].

There have been many research endeavors over the years focused on understanding the mechanisms of cisplatin nephrotoxicity. More recently, research efforts have focused more on understanding cellular and molecular events involved in renal susceptibility. Renal tubular epithelial cells selectively take up cisplatin, which induces mitochondrial dysfunction and an aberrant surge in ROS [70]. This event triggers stress response pathways and disrupts energy homeostasis, leading to apoptosis and/or necrosis.

Concurrently, a cisplatin-induced inflammatory response involves the release of pro-inflammatory cytokines. These cytokines contribute to increased tubular injury. Cisplatin also injures the microvascular components of the kidneys and impairs blood flow, leading to ischemic injury [91].

The nephrotoxicity induced by cisplatin is significantly dependent on dose, cumulative exposure, peak plasma concentration, and length of therapy. Optimizing dosing regimens via proper hydration, dosage fractionation, renal function-based dose modification, and therapeutic drug monitoring is crucial to reduce renal damage while preserving therapeutic effectiveness [92].

Importantly, cisplatin-induced nephrotoxicity exhibits a well-established dose- and exposure-dependent profile, with renal injury risk correlating strongly with cumulative dose, peak plasma concentration, and the number of treatment cycles [84,85]. Sustained intracellular accumulation of cisplatin within proximal tubular epithelial cells overwhelms mitochondrial antioxidant capacity, thereby amplifying oxidative stress, inflammatory signaling, and tubular cell death [83,89]. Consequently, clinical strategies aimed at optimizing dosing regimens, including adequate pre- and post-treatment hydration, dose fractionation, avoidance of high peak concentrations, and renal function-based dose adjustment, have been shown to attenuate nephrotoxicity while preserving antitumor efficacy significantly [85,89]. In this context, close monitoring of renal function, together with emerging tubular injury biomarkers, is critical for early detection of subclinical injury and for timely modification of cisplatin exposure [93,94].

**Figure 1:**
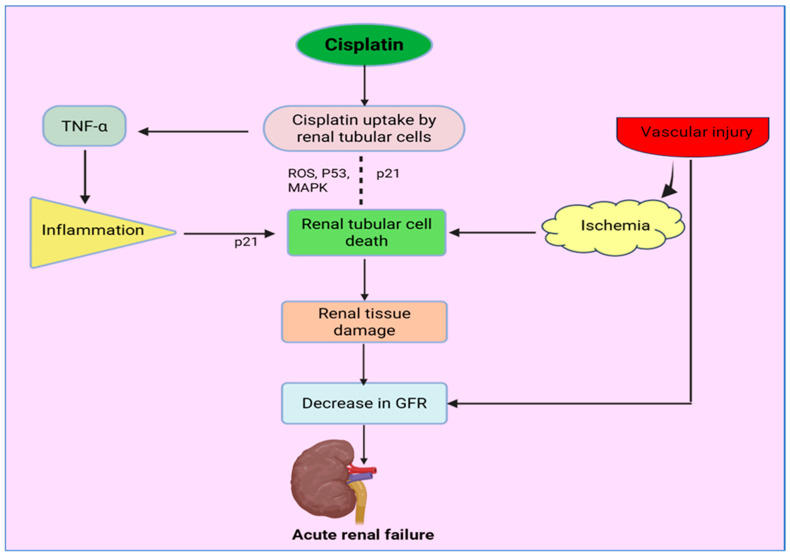
Schematic overview of the pathophysiological mechanisms underlying cisplatin-induced nephrotoxicity. Created in BioRender. NAAZ, N. (2026) https://BioRender.com/cy2bkq4 (accessed on 7 January 2026).

Taken together, these results highlight the complexity of cisplatin nephrotoxicity, which is no longer considered to involve the inhibition of a single pathway but rather results from multiple mechanisms: redox imbalances, amplification of inflammation, and hemodynamic changes. A summary of this is shown in Figure 1.

Cisplatin enters renal cells by passive diffusion and transport, leading to selective accumulation in tubular epithelial cells. The intracellular exposure to cisplatin disrupts mitochondrial function. It leads to an overproduction of ROS, triggering stress-activated signaling pathways such as MAPK and p53, ultimately causing apoptotic and necrotic cell death. Concurrently, cytoprotective pathways, such as p21, can be temporarily activated but are frequently overwhelmed by injury pathways. Cisplatin simultaneously triggers a substantial increase in inflammatory factors, especially tumor necrosis factor-α (TNF-α), adding further insult to tubular cells via inflammatory cell infiltration and cytokine action. Moreover, cisplatin-induced microvascular injury in the kidney impairs renal blood flow, causing ischemia and further increasing tubular cell mortality. The interplay of oxidative stress, inflammatory injury, and microvasculature impairment eventually results in a reduction in GFR and a consequent event of acute kidney injury.

#### 4.1.2. Cyclophosphamide

Cyclophosphamide is a prodrug that undergoes hepatic biotransformation to become active. The active derivatives of cyclophosphamide produced during biotransformation in the liver include phosphoramide mustard and acrolein [95]. These two derivatives contribute to the compound’s mechanism of action and toxicity. The mechanism of action involves increased ROS formation, leading to low antioxidant levels in the system and the kidneys [96].

Among cyclophosphamide metabolites, acrolein is a major contributor to renal injury. Acrolein is a highly reactive aldehyde that promotes oxidative stress by directly generating free radicals and by depleting intracellular glutathione (GSH) through covalent binding. Loss of GSH compromises glutathione-dependent antioxidant defenses, thereby amplifying redox imbalance and facilitating further ROS accumulation [64]. In addition, acrolein disrupts nitric oxide signaling in the renal vasculature, contributing to endothelial dysfunction and increased oxidative burden by dysregulating the nitric oxide/nitric oxide synthase axis [97].

Cyclophosphamide-induced nephrotoxicity is associated with tubular dysfunction and a decline in glomerular filtration rate, which are consequences of direct tubular injury and, in particular, the resulting vascular changes [97]. The key downstream effect of oxidative stress is lipid peroxidation. Acrolein-mediated peroxidation of cell membrane lipids generates malondialdehyde, a commonly used biomarker of oxidative stress that can further compromise cell membrane integrity and renal cell function [98].

At the molecular level, cyclophosphamide toxicity results from two highly interrelated mechanisms. First, the depletion of nucleophilic antioxidants, such as glutathione, makes renal cells more susceptible to oxidative stress. Second, overproduction of reactive nitrogen species occurs when nitric oxide reacts with superoxide, yielding peroxynitrite, a highly toxic oxidant. The production of peroxynitrite in regions of inflammation leads to the oxidation of lipids and proteins, thereby potentiating cellular and tissue dysfunction [99]. Activated inflammatory cells, such as neutrophils, monocytes, and macrophages, also contribute to ROS production, thereby perpetuating the vicious cycle between oxidative stress and inflammation [100].

Overall, the cumulative effect of cyclophosphamide overwhelms the kidney’s antioxidant defense mechanisms, leading to redox imbalances and the activation of inflammatory pathways, ultimately resulting in apoptosis and necrotic cell death [101]. The cumulative effects of oxidative and inflammatory stresses result in structural remodeling, leading to functional loss and, in some cases, renal failure [102,103]. Optimization of dosing through renal function-based dose adjustment, adequate hydration, and regular monitoring of renal function and biomarkers is essential to minimize nephrotoxicity [104]. To avoid nephrotoxicity and preserve effectiveness, cumulative dosage and therapy duration must be monitored clinically.

Like other alkylating agents, cyclophosphamide-associated nephrotoxicity is closely linked to cumulative dose and duration of exposure, particularly in high-dose or prolonged treatment regimens [97]. Progressive accumulation of toxic metabolites, such as acrolein, exacerbates oxidative stress by depleting glutathione and promoting lipid peroxidation, thereby lowering the threshold for tubular and vascular injury [98]. Accordingly, optimizing cyclophosphamide dosing through renal function-guided dose adjustment, maintaining adequate hydration, and regularly monitoring renal function and injury biomarkers are essential to limit dose-dependent renal damage [102,103]. These strategies are especially relevant in patients with pre-existing renal impairment or concomitant exposure to other nephrotoxic agents, in whom cumulative oxidative burden may accelerate kidney injury.

**Figure 2:**
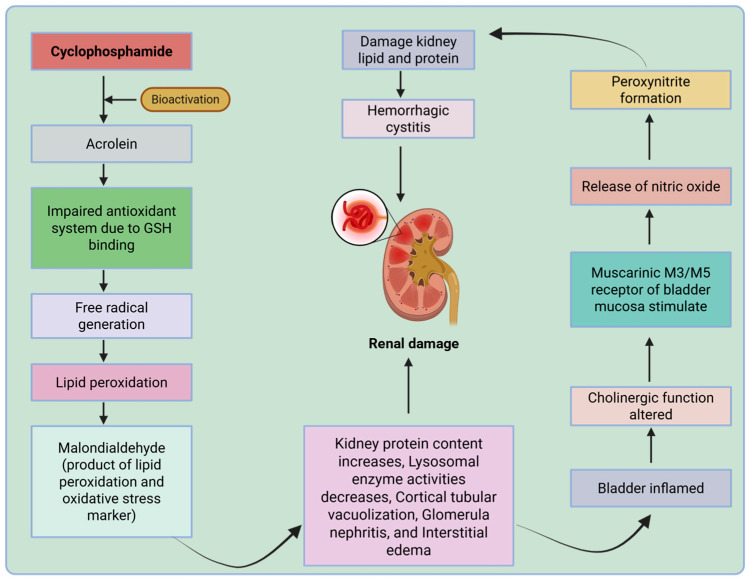
Schematic representation of the mechanisms underlying cyclophosphamide-induced nephrotoxicity and their clinical manifestations. Created in BioRender. NAAZ, N. (2026) https://BioRender.com/cy2bkq4 (accessed on 7 January 2026).

The key oxidative and inflammatory pathways mediating cyclophosphamide-induced nephrotoxicity are depicted in Figure 2.

Nephrotoxic agents disrupt renal tubular and glomerular function, leading to abnormalities in urine sediment, electrolyte disturbances, and—most prominently—a reduction in GFR. These functional alterations arise from the combined effects of tubular epithelial injury, oxidative stress, inflammatory signaling, and impaired renal hemodynamics, ultimately contributing to the development of acute kidney injury [79].

### 4.2. Antibiotics

Aminoglycoside antibiotics, such as gentamicin, tobramycin, amikacin, netilmicin, neomycin, and streptomycin, are commonly utilized in clinical settings for the treatment of serious infections caused primarily by Gram-negative bacteria and certain Gram-positive organisms [105]. Despite their clinical efficacy, aminoglycosides are well recognized for their nephrotoxic potential. Renal injury associated with these agents arises through a combination of tubular cytotoxicity, altered renal hemodynamics, and mesangial cell dysfunction, with direct tubular epithelial injury representing the dominant mechanism [106].

Gentamicin and related aminoglycosides induce both apoptosis and necrosis in renal tubular epithelial cells, as demonstrated in cell culture systems and experimental animal models [86]. Following glomerular filtration, aminoglycosides are taken up by proximal tubular cells via endocytic pathways and accumulate within intracellular organelles, including lysosomes, the Golgi apparatus, and the endoplasmic reticulum. At intracellular concentrations above a critical threshold, these drugs enter the cytosol and interact with mitochondria, leading to mitochondrial dysfunction, excessive reactive oxygen species production, and activation of cell death pathways [107].

In addition to promoting tubular cell death, aminoglycoside antibiotics can impair renal tubular function by inhibiting membrane transporters responsible for solute reabsorption, thereby affecting electrolyte and glucose reabsorption [108]. The drugs can also induce mesangial cell contraction and increase intracellular calcium, thereby affecting glomerular filtration dynamics [109]. Oxidative stress is a significant mediator of these pathological processes, which induce cell damage and interfere with adaptive repair.

Aminoglycosides also influence renal hemodynamic changes. Vasoactive substances such as platelet-activating factor, endothelin-1, and thromboxane A2 are released from the cells of the endothelium and the mesangium in response to the drug [110]. These vasoactive agents induce vasoconstriction of the kidneys’ microvessels via paracrine mechanisms, thereby contributing to reductions in renal blood flow and glomerular filtration rate [87,111].

All the aforementioned renal injuries associated with aminoglycosides contribute to the progression of renal impairment and dysfunction.

**Figure 3:**
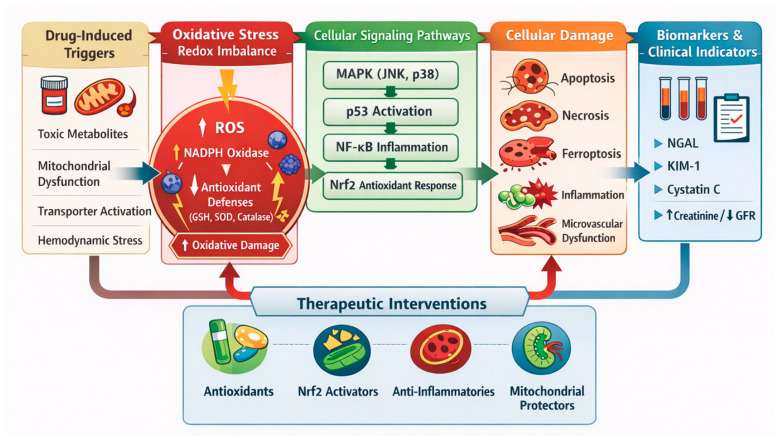
Drug-specific molecular initiation and convergence of oxidative stress-mediated nephrotoxicity. Nephrotoxic drugs initiate renal injury through distinct molecular events, including differential cellular uptake, metabolic activation, or primary mitochondrial perturbation. These initiating processes activate upstream signaling nodes—such as p53-mediated mitochondrial stress responses—that subsequently converge on shared redox-sensitive pathways. The integrated activation of oxidative stress, inflammatory signaling, and regulated cell death culminates in tubular and vascular dysfunction, representing a final common pathway of drug-induced kidney injury. Created in BioRender. NAAZ, N. (2026) https://BioRender.com/cy2bkq4 (accessed on 7 January 2026).

Clinically, aminoglycoside-induced nephrotoxicity is manifested as proteinuria, glycosuria, and electrolyte disturbances, such as hypokalemia, hypocalcemia, and hypomagnesemia, reflecting impairment of proximal tubular function. The major cellular and vascular mechanisms of aminoglycoside-induced renal injury are schematically represented in Figure 3.

Although drug-induced nephrotoxicity converges on shared downstream processes, including oxidative stress, inflammation, and cell death, these events are initiated by distinct drug-specific molecular mechanisms. For example, cisplatin enters proximal tubular cells primarily via organic cation transporter 2, where intracellular accumulation triggers mitochondrial DNA damage and excessive ROS production; tacrolimus induces oxidative injury predominantly through calcineurin inhibition-associated mitochondrial dysfunction and vasoconstriction; cyclophosphamide generates nephrotoxic metabolites such as acrolein that directly deplete antioxidant defenses; and aminoglycosides such as gentamicin accumulate in lysosomes, leading to phospholipidosis, mitochondrial stress, and secondary ROS amplification [86,89,98]. These drug-specific initiating events activate discrete upstream signaling nodes, including p53, MAPK, NF-κB, and redox-sensitive mitochondrial pathways, before converging on the final common pathways depicted in Figure 3. Recognition of these molecular entry points provides a mechanistic basis for identifying targeted preventive and therapeutic strategies tailored to specific drug classes.

### 4.3. Antiviral Drugs

Antiviral drugs play an important role in treating various viral infections, but they often cause nephrotoxicity due to their preferential renal uptake [33]. Various antiviral drugs tend to have affinity for proximal tubular cells in the kidneys; hence, the region is particularly sensitive to their toxicity. The renal toxicity induced by various antiviral drugs is often in the form of acute renal injury, tubular dysfunction, or even chronic kidney disease [112].

Cidofovir, a nucleotide analog active against DNA viruses including cytomegalovirus, herpes simplex virus, and human papillomavirus, is widely used to treat CMV retinitis in patients with AIDS [113]. One of the dose-limiting side effects of cidofovir is its nephrotoxicity, which is associated with acute renal failure and proximal tubule cell injury. Experimental studies have shown that cidofovir induces apoptosis in tubular cells, thereby directly causing tubular dysfunction and renal dysfunction [83].

Adefovir, a further nucleotide analog with therapeutic activity against human immunodeficiency virus and Hepatitis B virus infections, shares a similar nephrotoxicity profile [114]. Prolonged treatment with adefovir has been linked with the development of AKI and Fanconi-like syndrome, which suggests selective tubular toxicity of proximal tubular cells [115,116]. Cidofovir and adefovir are substrates for organic anion transporter 1 (OAT1), which facilitates their entry into tubular cells and increases their toxicity [117].

Recent findings also suggest that mitochondrial toxicity appears to play an important role in the nephrotoxicity associated with such drugs. It has been found that mitochondrial DNA depletion, mitochondrial oxidative phosphorylation dysfunction, and disturbances in cellular energy balance induced by cidofovir and adefovir contribute to mitochondrial toxicity and tubular cell death through oxidative stress [118].

Tenofovir, a nucleoside reverse transcriptase inhibitor structurally similar to cidofovir and adefovir, has been widely used to manage HIV and hepatitis B infections and, unlike its predecessors, is perceived to have a more favorable renal safety profile [119]. However, its perceivably lower incidence of nephrotoxicity has already been followed by an association with AKI and chronic kidney disease, as reported in various clinical cases [120]. Though the mechanism remains poorly understood, emerging evidence among researchers points to mitochondrial dysfunction due to inefficient tubular energy metabolism, suggesting that nucleotide analog antiviral medications may share pathogenic mechanisms [121].

In contrast, acyclovir, an antiviral agent used primarily to treat herpesvirus infections, induces renal toxicity primarily through crystal-induced tubular obstruction rather than direct cellular toxicity [122]. The majority of acyclovir is excreted through the kidneys in an unchanged form, and limited solubility in the urine leads to intratubular crystallization, especially in conditions of dehydration or high-dose therapy [123]. Obstructive tubulopathy due to acyclovir crystallization may lead to acute renal failure, often accompanied by metabolic disturbances, including bicarbonate abnormalities, electrolyte disturbances, proteinuria, and glycosuria [70].

Taken together, antiviral medication-induced nephrotoxicity demonstrates a convergence of tubular accumulation, mitochondrial damage, oxidative injury, and, in certain instances, intratubular crystals. The mechanisms are well understood, and it is clear that dose optimization, hydration, and redox protection strategies play important roles in preventing renal damage during antiviral therapy.

### 4.4. Nonsteroidal Anti-Inflammatory Drugs (NSAIDs)

Nonsteroidal anti-inflammatory drugs, or NSAIDs, are amongst the most commonly prescribed and over-the-counter dispensed medications worldwide and have long been employed for acute and chronic pain, as well as for inflammation and, in particular, for reducing fever [124]. The global consumption level of NSAIDs has increased during the past decades with the over-the-counter distribution of these medications, and, as a result, there has been greater concern over renal side effects from NSAIDs.

NSAIDs work by inhibiting cyclooxygenase (COX) activity, thereby reducing prostaglandin synthesis. Based on their selectivity, NSAIDs can be broadly classified into COX-1-selective inhibitors (e.g., aspirin), COX-2-selective inhibitors (e.g., celecoxib, meloxicam), and non-selective COX inhibitors (e.g., ibuprofen, diclofenac, naproxen, ketoprofen) [125]. Whereas COX-2-selective inhibitors were initially developed to minimize gastrointestinal toxicity associated with COX-1 inhibition, studies have since identified widespread expression of COX-2 in the kidney, including the proximal tubules, renal endothelium, and vasculature. This has meant that COX-2 inhibition similarly impairs renal function, comparable with non-selective NSAIDs.

Renal prostaglandins are very important in maintaining renal blood flow and glomerular filtration rate, especially in situations of reduced effective circulating volume or renal hypoperfusion [126]. The mechanism of NSAIDs in reducing prostaglandin synthesis, causing vasoconstriction of the afferent arteriole, decreased renal perfusion, and subsequent decline in glomerular filtration rate, puts susceptible patients, especially the elderly, volume-depleted, and those with pre-existing renovopathies, at risk for acute renal failure.

Clinically, NSAID nephrotoxicity can present with oliguric acute renal failure, electrolyte imbalances such as mild water intoxication or hypokalemia, proteinuria, volume expansion, and hypertension. In addition, some patients with NSAID nephrotoxicity have exhibited acute interstitial nephritis and papillary nephrocalcinosis or papillary necrosis. In addition, a reduction in renal blood flow could, in turn, increase oxidative stress and promote an inflammatory response, further accentuating tubular and interstitial injury.

To prevent renal toxicity induced by NSAIDs, certain measures have been proposed [127]. These include avoiding a combination of analgesic drugs in a single patient as a possible source of renal problems and avoiding prolonged use of NSAIDs. Furthermore, caution should be observed in certain risk groups. Early diagnosis and immediate stoppage of NSAID use remain fundamental in preventing progression of renal injury to an irreversible stage.

### 4.5. Immunosuppressant

Calcineurin inhibitors, the major ones being cyclosporine A and tacrolimus, are keystone immunosuppressive drugs used to prevent allograft rejection in solid organ transplantation. Despite their clinical potency, nephrotoxicity remains a major defect associated with their long-term application [88]. The incidence of calcineurin inhibitor-induced renal dysfunction is widely quoted, with some estimates suggesting an incidence of 18–42% alone in liver transplant recipients [128].

Nephrotoxicity induced by Cyclosporine A occurs mainly through vasoconstriction in the kidneys, leading to decreases in renal blood flow and glomerular filtration rate [129]. Experimental investigations have revealed that Cyclosporine A induces vasoconstriction in glomerular arterioles, thereby causing ischemic damage to renal tissues [82]. Furthermore, in injured kidneys, Cyclosporine A has been observed to augment intrarenal renin and simultaneously depress the production of vasodilatory prostaglandins, thus superimposing further on hemodynamic abnormalities [130]. Initially, such abnormalities are readily reversible with dose reduction or treatment cessation, but they may eventually cause irreversible damage [131].

Tacrolimus is similar to cyclosporine in its mechanism of action, through the inhibition of calcineurin, but is more potent and is associated with fewer adverse reactions outside the renal system when considering cyclosporine A [132]. However, Tacrolimus induces vasoconstriction in both the efferent and afferent arterioles of the glomerulus, which, in turn, compromises renal perfusion and the GFR through tubular necrosis due to ischemia. This is particularly so with sustained hemodynamic forces, ultimately leading to oxidative nephron injury.

Interindividual variability in tacrolimus pharmacokinetics is a critical determinant of nephrotoxicity risk. Tacrolimus undergoes extensive hepatic metabolism primarily via cytochrome P450 3A enzymes and is transported by P-gp, encoded by the *ABCB1* gene [133]. Genetic polymorphisms in genes encoding the CYP3A isoenzymes, particularly CYP3A5, significantly impact drug clearance and systemic exposure. CYP3A5 expression is associated with higher tacrolimus clearance and the need for higher doses to attain therapeutic concentrations [134]. However, dose escalation in these patients will increase the risk of chronic nephrotoxicity, particularly with additional nephrotoxic stressors [135,136].

Nephrotoxicity caused by cyclosporine occurs through two different mechanisms. Acute nephrotoxicity is mostly reversible and involves hypertension, decreased GFR, and RPF. However, chronic nephrotoxicity is mostly irreversible [137]. The signs of this condition include chronic hypertension, mild proteinuria, increased interstitial fibrosis, and a reduction in GFR [138]. Preventive strategies to reduce renal insults caused by calcineurin inhibitors include reducing the calcineurin inhibitor dose, using a calcium channel blocker or a prostaglandin analog such as misoprostol, and avoiding potential interactions. It is important to carefully consider the use of drugs such as verapamil, anabolic steroids, ranitidine, and cimetidine due to their potential ability to modulate the metabolism of the calcineurin inhibitors [139]. The main vascular and tubular changes responsible for the nephrotoxicity associated with the use of calcineurin inhibitors are shown in Figure 3.

### 4.6. Contrast Agents

These contrast agents are used indiscriminately in diagnostic and interventional imaging studies to enhance visualization of vascular structures and soft tissues. Although clinically useful, contrast-induced nephropathy remains a leading cause of hospital-acquired AKI, accounting for approximately 11–12% of AKI cases in hospitalized patients [140]. Renal dysfunction following the administration of contrast typically presents as an increase in serum creatinine within 24–48 h, peaks on days two to three, with a gradual recovery to baseline renal function within one to two weeks in most instances [141].

The underlying mechanisms of contrast-induced nephrotoxicity are complex and occur through a complex series of renal hemodynamic changes, oxidative damage, and direct tubular toxicity [142]. There may be hypoxia of the renal medulla due to reduced renal blood flow and increased oxygen utilization, specifically in the outer medullary region of the renal cortex. This may occur concurrently with increased ROS production, leading to mitochondrial dysfunction and tubular epithelial damage. Other factors may involve adenosine-induced vasoconstriction and impaired endothelial function [143,144].

Contrasting agents can be broadly categorized by plasma osmolality as high-osmolality, low-osmolality, or iso-osmolality agents [145]. The higher the osmolality, the greater the osmotic stress, with iso-osmolality agents being closer to physiological values. Evidence shows that iodixanol, an iso-osmolar non-ionic iodinated contrast agent, is less nephrotoxic than low-osmolar agents such as iohexol in high-risk patients with renal impairment undergoing vascular angiography procedures. Higher osmotic stress increases the kidneys’ oxygen demands and creates an oxygen consumption–delivery mismatch, increasing vulnerability to medullary damage.

Importantly, the development of contrast-induced nephrotoxicity depends not only on osmolality but also on viscosity and ionic properties of the contrast medium [146]. The increased viscosity of the contrast agent in the renal tubules increases intratubular pressure, decreases tubular flow, and impairs medullary perfusion, thereby further enhancing hypoxia and oxidative stress [147]. These physicochemical factors, together, are responsible for tubular obstruction, endothelial dysfunction, and decreased glomerular filtration.

To prevent contrast-induced renal injury, measures such as the preferential use of non-nephrotoxic contrast media, minimizing contrast volume, and using imaging techniques that do not require contrast media, especially in those at risk of renal dysfunction, can be employed. Early identification of susceptibility and optimization of renal perfusion are fundamental in nephroprotection.

Nephrotoxic drugs such as cisplatin, cyclophosphamide, tacrolimus, and gentamicin enter renal cells via passive diffusion and facilitated uptake, leading to preferential accumulation in tubular epithelial cells. Cisplatin damages DNA and mitochondria, triggering p53-mediated apoptosis and secondary necrosis, whereas cyclophosphamide generates hazardous metabolites that induce oxidative stress and apoptosis, leading to necrosis. Tacrolimus promotes ischemia and mitochondrial dysfunction, causing apoptosis. Gentamicin accumulates in lysosomes and mitochondria, causing oxidative damage, membrane disruption, and combined apoptotic–necrotic tubular injury [148].

Intracellular drug concentrations disrupt mitochondrial function, leading to excessive ROS production and activation of stress-related signaling cascades, such as MAPK and p53, ultimately contributing to apoptotic and necrotic cell death [149]. Concurrent with cellular activation, protective signaling pathways, such as p21, may be transiently activated, but this is often futile. Drug-related production of the pro-inflammatory mediator TNF-α exacerbates tubular injury through inflammation-related signaling and immune system recruitment. Additionally, vascular injury and vasoconstriction reduce renal perfusion, leading to ischemia, tubular cell injury, and a subsequent reduction in glomerular filtration rate [150]. Ultimately, the convergence of ROS, inflammation, and hemodynamic dysfunction is reflected in clinical presentation with changed urine sediment characteristics, electrolyte imbalance, and acute renal failure.

## 5. Biomarkers for Assessment of Drug-Induced Nephrotoxicity

Precise estimation of renal function is important in the clinical setting and during drug safety assessment. The GFR is the most valid marker of overall renal function and is predominantly used for staging renal disease and measuring its progression [151,152]. The vast majority of methods currently used for GFR estimation are bulky and cumbersome and thus do not apply to nephrotoxicity detection.

In clinical practice, serum creatinine and blood urea nitrogen (BUN) are the most common biomarkers of renal function [153]. These endogenous waste products are mainly excreted via the kidneys and accumulate in the circulation due to renal injury. Estimation of GFR from plasma creatinine concentration is widely used; however, its accuracy is influenced by several non-renal factors, including age, sex, muscle mass, diet, and body composition [154]. For this reason, measurements based on creatinine frequently fail to detect early or mild renal injury and may poorly estimate the extent of kidney dysfunction.

Direct measurement of renal clearance by exogenous filtration markers, including inulin, iothalamate, and technetium-99m–diethylenetriaminepentaacetic acid (^99m^Tc-DTPA), provides a more accurate determination of GFR but is invasive and resource-intensive [155]. These approaches entail repeated blood or urine sampling and cumbersome analytical techniques, such as high-performance liquid chromatography. Further compromising interpretive accuracy, emerging evidence indicates that proximal tubular secretion of creatinine and iothalamate, as well as tubular reabsorption of cystatin C and ^99m^Tc-DTPA, may introduce systematic bias into direct GFR measurements [156,157].

Nonetheless, recent breakthroughs have enabled the development of transcutaneous fluorescence-based methods for the noninvasive, real-time assessment of renal function [151]. These involve the use of fluorescence-based markers, including Near-Infrared (NIR) fluorescent dyes with enhanced tissue penetration, for fast, noninvasive estimation without requiring blood or urine collection [158]. Though these methods make it easier to measure the GFR, they are only useful for detecting functional damage after substantial nephron loss and do not provide the spatial resolution to discriminate segmental renal damage. Thus, any function-based method, regardless of the approach, is an indirect late indicator of nephrotoxicity [159].

Indeed, since conventional markers of renal function are only sensitive to renal injury once substantial filtration functionality is lost, clinically significant nephrotoxicity induced by various medications is frequently diagnosed at a relatively late stage and with restricted potential for early intervention [33]. This relative deficiency notwithstanding, attention has recently focused on identifying more sensitive and specific markers of renal injury that can detect it at an earlier, reversible stage. Within the past decade, novel biomarkers of high levels of both serum and urinary reactivity are highly specific for renal cellular toxicity, including neutrophil gelatinase-associated lipocalin (NGAL) and clusterin, as well as enzymatically active urinoproteins such as N-acetyl-β-D-glucosaminidase (NAG) [160].

Given the limited specificity of individual biomarkers, a panel-based strategy is increasingly recognized as essential for accurate characterization of drug-induced nephrotoxicity [130,132]. The coordinated assessment of functional markers, together with site- and mechanism-specific injury biomarkers, enables discrimination between glomerular, tubular, and interstitial damage, as well as the identification of dominant cell death pathways [134,140]. For example, early elevations in tubular injury markers such as kidney injury molecule-1 (KIM-1) and neutrophil gelatinase-associated lipocalin (NGAL), followed by increases in mitochondrial dysfunction or lipid peroxidation markers, are indicative of proximal tubular injury driven by oxidative stress [133,144]. In contrast, concurrent changes in cystatin C levels or albuminuria are more suggestive of glomerular involvement [135,138]. Moreover, the temporal sequence and relative magnitude of apoptosis-associated markers, necrosis-related damage-associated molecular patterns (DAMPs), and ferroptosis-linked lipid peroxidation products provide mechanistic insight into the prevailing mode of cell death [47,143]. Adoption of such multi-marker panels, therefore, provides a more robust framework for toxicity classification, mechanistic interpretation, and clinical decision-making in nephrotoxic drug exposure.

Although biomarkers such as NGAL, KIM-1, and cystatin C show promise for early detection of drug-induced kidney injury, their clinical implementation remains limited by variability in sensitivity and specificity across injury types, lack of standardized cutoff values, and incomplete discrimination between primary oxidative injury and secondary inflammatory or hemodynamic processes.

Regarding this subject, the contemporary review focuses on both conventional and novel biomarkers used to diagnose drug-induced renal toxicity.

### 5.1. Kidney Injury Molecule-1 (KIM-1)

Kidney injury molecule-1 (KIM-1) is a transmembrane type I glycoprotein with a mucin-rich domain and an immunoglobulin-like domain, where there is a conserved cysteine motif of six cysteines [161]. KIM-1 has low expression in normal kidneys but is highly induced in proximal tubular cells following renal injury; it is therefore a sensitive marker [162].

Several key points distinguish KIM-1 from other markers of renal injury. Firstly, the rapid increase in KIM-1 in plasma and urine, evident as early as 2–4 h post-renal injury, precedes the rise in serum creatinine, BUN, and NAG, establishing its sensitivity to injury [163,164]. Secondly, KIM-1 expression is injury type-specific and reflects proximal tubular injury rather than the overall reduction in filtered mass. Finally, KIM-1 is an exhaustive renal protein and is abundantly expressed in ischemic injury, renal cell carcinoma, and polycystic kidney disease. Notably, the timing of KIM-1 measurement affects diagnostic accuracy. A meta-analysis of KIM-1 measurements in urine 2–6 h post-cardiopulmonary bypass showed a high sensitivity of more than 90% compared with measurements at 12 h, which had a lower sensitivity of around 74% [165].

Experimental evidence shows that KIM-1 mRNA and protein expression are significantly induced after ischemic and toxic renal injury, whereas they are barely detectable under normal conditions [166,167]. Indeed, experimental models of nephrotoxicity induced by various drugs, such as cisplatin, cyclosporine, and gentamicin, have all shown a significant rise in KIM-1 excretion into the urine before an increase in serum creatinine, blood urea nitrogen, or proteinuria, and before glycosuria [168]. For example, cisplatin administration induces a three- to five-fold increase in urinary KIM-1 without concurrent changes in traditional renal biomarkers. Similarly, exposure to nephrotoxicants such as S-(1,1,2,2-tetrafluoroethyl)-L-cysteine, folic acid, and cisplatin results in pronounced KIM-1 expression within proximal tubular epithelial cells [169]. Comparative toxicology studies have further shown that KIM-1 outperforms serum creatinine, BUN, and NAG in detecting early acute kidney injury induced by gentamicin, mercury, and chromium [170].

Transcriptomic studies confirm the diagnostic value of KIM-1. In studies analyzing differences between nephrotoxic and hepatotoxic substances, KIM-1 was identified as the gene with the highest induction among renal-specific genes, together with osteopontin and lipocalin-2 (Lcn2), and was found to be associated only with proximal tubular necrosis. It is important to note that KIM-1 was not induced to the same extent by hepatotoxic substances [170,171].

Although more limited, clinical evidence supports the translational utility of KIM-1 as an early biomarker of drug-induced nephrotoxicity. In patients receiving cisplatin chemotherapy, urinary KIM-1 levels consistently rise before detectable changes in serum creatinine [93]. In a cohort of lung cancer patients, urinary KIM-1 and monocyte chemoattractant protein-1 (MCP-1) demonstrated superior diagnostic accuracy for cisplatin-induced acute kidney injury compared with NGAL, NAG, or β2-microglobulin. Other longitudinal studies have reported sustained elevations in urinary KIM-1 following cisplatin exposure, although its ability to discriminate between patients with and without clinically overt acute kidney injury remains variable [94,172,173]. In neonatal populations, urinary KIM-1 has also been shown to increase following gentamicin exposure, indicating sensitivity across age groups [174].

In conclusion, the presence of KIM-1 in urine is recognized as the most reliable and sensitive indicator for the early assessment of proximal tubular damage and, more importantly, for the assessment of nephrotoxicity induced by drugs. This is because KIM-1 is significantly elevated before the conventional marker of renal damage. However, limitations include variability in optimal sampling times, limited assay standardization, and limited validation in large, diverse patient populations.

### 5.2. Proteinuria and Albuminuria

Under physiological conditions, the excretion of albumin in urine is low; it normally does not exceed 30 mg/g of creatinine [175]. The albumin present in blood is a high-molecular-weight protein with a molecular weight of approximately 66.5 kDa; its excretion in urine indicates a disturbance in renal barrier integrity [176]. Both glomerular and tubular dysfunction can lead to proteinuria, although the degree of albumin excretion can vary depending on whether glomeruli or tubules are primarily affected. Tubular dysfunction is more likely to be associated with a lower degree of albuminuria, which may be <500 mg/24 h because of a deficiency of tubular reabsorptive capacity rather than glomerular permeability [177].

Proteinuria and albuminuria have been widely tested as biomarkers of drug-induced nephrotoxicity, most often in patients undergoing cisplatin-based chemotherapy. In a group of 57 patients treated with cisplatin, urinary albumin rose approximately twofold by day 10 after treatment, without overt acute kidney injury [173]. Another study of 33 patients treated with cisplatin showed that albuminuria rose to 5.6 times control levels by day 4 among patients who developed AKI. In contrast, it rose to 3.4-fold baseline levels by day 3 in those who did not develop AKI [178]. Other studies have also demonstrated marked increases in albuminuria after repeated exposure to cisplatin.

Additional evidence of tubular dysfunction has been found in the assessment of high-dose cisplatin exposure [92]. In patients treated with multiple cycles of cisplatin at a dose of 40 mg/m^2^ daily for 5 consecutive days, enhanced excretion of β2-microglobulin (B2M), NAG, and amino acids, along with albuminuria and total proteinuria, was observed [179]. The data point to simultaneous glomerular and tubular dysfunction post-cisplatin exposure, although it is unclear to what extent enhanced proteinuria and albuminuria can predict the onset of clinically evident acute kidney injury, as assessed by serum creatinine levels and/or the need for renal replacement therapy.

Besides cisplatin, cyclosporine A has also been reported to produce proteinuria and albuminuria in animal studies and human patients. In animal studies, prenatal and long-term exposure to cyclosporine has been associated with lower nephron number, higher blood pressure, and higher albuminuria in exposed groups compared with controls [180]. In 21 days of cyclosporine (20 mg/kg/day) treatment in Wistar male rats, a higher serum creatinine concentration and increased urinary albumin-to-creatinine ratios were observed compared with controls [181]. Indeed, proteinuria induced by cyclosporine in animal studies has long been recognized as an indicator of calcineurin inhibitor-induced nephrotoxicity [182].

In conclusion, albuminuria reflects greater glomerular involvement than tubular damage and is a frequent manifestation of nephrotoxicity caused by agents such as cisplatin and cyclosporin. Although elevated levels of albuminuria have been documented both in experimental and clinical studies, their use as predictors of severe acute kidney injury has remained inaccurate. Therefore, albuminuria is best measured simultaneously with other markers of renal injury to assess nephrotoxicity.

### 5.3. Neutrophil Gelatinase-Associated Lipocalin (NGAL)

Neutrophil gelatinase-associated lipocalin, or NGAL, is a 21 kDa protein of the lipocalin family and an important innate immune factor because of its binding affinity to iron-loaded siderophores during infections and inflammation [183]. In pathological conditions, NGAL mRNA and peptide expression are upregulated in various cell types, including immune cells, hepatocytes, and renal tubular cells. There is relative resistance of the polypeptide to enzymatic cleavage and ease of passage through the urine, making it possible to be detected in biological fluids [184].

Experimental studies have clearly shown a significant induction of NGAL gene expression in renal and urinary samples after ischemic renal injury [185]. In a murine model of ischemic renal injury, urinary NGAL appears earlier than increases in other conventional urinary biomarkers, such as NAG or B2M. Human studies confirm a significant increase in urinary NGAL concentrations as early as 6 h after a renal insult during cardiopulmonary bypass in adults and children [186,187]. As a result, NGAL has emerged as one of the most extensively studied novel biomarkers across a wide range of clinical and experimental settings.

The utility of NGAL as a biomarker for cisplatin-induced nephrotoxicity has been extensively evaluated. In preclinical studies, increased NGAL expression has been detected in the kidneys of mice during the early phase of cisplatin-induced injury [188]. In human studies, urinary NGAL has been proposed as an early indicator of cisplatin-associated AKI. A systematic evaluation of nine clinical investigations assessing urinary NGAL in patients receiving cisplatin reported that six studies observed elevated NGAL levels shortly after treatment, regardless of whether patients developed clinically apparent AKI. However, the predictive performance of NGAL varied, with reported areas under the receiver operating characteristic (ROC) curve ranging from 0.6 to 0.8, reflecting moderate diagnostic accuracy [189].

NGAL has also demonstrated sensitivity as an early marker of aminoglycoside-induced nephrotoxicity in animal models. In male Sprague–Dawley rats treated with gentamicin for 1, 3, or 7 days, renal and urinary NGAL levels increased in a dose- and time-dependent manner, paralleling changes in KIM-1. Most importantly, these changes occurred before histopathological injury and before an increase in serum creatinine levels [190]. Similar observations have been made in dogs and mice, in which urinary NGAL levels have been used as highly sensitive and non-invasive biomarkers of gentamicin-induced acute kidney injury [191,192].

Beyond cisplatin and aminoglycosides, urinary NGAL has been studied in the setting of other nephrotoxic agents. In subjects treated with amphotericin B, urinary NGAL rose approximately 3 days before serum creatinine, suggesting a temporal advantage for early detection of renal injury. Collectively, these results demonstrate that NGAL is a responsive biomarker of tubular injury across diverse forms of nephrotoxicity. Summary: Urinary NGAL is an early, noninvasive biomarker for detecting drug-induced kidney injury, especially in experimental models and selected clinical contexts [193]. However, variability in diagnostic performance across patient populations underscores the need for larger, well-controlled human studies to validate its clinical utility, especially when used in isolation. Combining NGAL with complementary injury-specific biomarkers may improve sensitivity and specificity for early detection of nephrotoxicity [194]. However, its clinical specificity is limited, as systemic inflammation, infection, and cancer can raise levels. Assay standardization and validation across varied clinical contexts are important challenges for routine use [195].

### 5.4. Urinary Proteins with Enzymatic Activity

Several enzymes derived from renal tubular epithelial cells are released into the urine following cellular injury and have been used as biomarkers of acute and chronic drug-induced kidney damage [196]. These enzymatic markers reflect loss of tubular integrity rather than changes in glomerular filtration and therefore provide complementary information to functional biomarkers. Commonly studied urinary enzymes include alanine aminopeptidase, alkaline phosphatase, glutathione S-transferase, γ-glutamyl transpeptidase, and NAG [197].

Among these, NAG is one of the most extensively investigated enzymatic biomarkers. NAG is a high-molecular-weight (~140 kDa) lysosomal enzyme predominantly localized within proximal tubular epithelial cells [198]. Under normal physiological conditions, its large size prevents glomerular filtration; thus, its presence in urine reflects tubular cell injury and lysosomal leakage rather than altered filtration [199]. For several decades, urinary NAG has been employed as a diagnostic indicator of proximal tubular dysfunction, and accumulating evidence supports its sensitivity for detecting early tubular injury [200].

Elevated urinary NAG levels have been reported in experimental models exposed to a variety of nephrotoxic agents, including contrast media and aminoglycoside antibiotics, as well as in patients with renal disorders such as glomerulonephritis, chronic pyelonephritis, and hypertensive nephrosclerosis [201]. These findings underscore NAG’s broad responsiveness to tubular injury across diverse pathological contexts.

In addition to its diagnostic role in urine, NAG is recognized for its prognostic value. Indeed, in a prospective group of hospitalized patients with a diagnosis of acute renal failure, the level of the urine NAG is highly correlated with the severity of disease measures such as the APACHE II score, the need for renal replacement therapy, and in-hospital deaths [202]. More importantly, it was found that in-hospital deaths were significantly increased in patients with severely increased excretion of the NAG compared with the outcome in patients with lower excretion levels [203].

Despite its sensitivity, urinary NAG does not present specificity about the cause of renal injury and, in fact, can be raised in both toxic and non-toxic renal disorders. Consequently, its greatest clinical value lies in combination with injury-specific and mechanistic biomarkers, rather than as a standalone indicator. Future efforts to define the prognostic and predictive utility of enzymatic urinary biomarkers should focus on large, multicenter clinical studies integrating enzymatic markers with emerging molecular and redox-sensitive biomarkers.

### 5.5. Beta-2 Microglobulin (B2M)

Beta-2 microglobulin (B2M) is a low-molecular-weight protein (~11 kDa) that forms an integral component of major histocompatibility complex (MHC) class I molecules and is produced by most nucleated cells, with particularly high expression in activated lymphocytes [155]. Under pathological conditions associated with immune activation—such as infection, autoimmune disease, or certain malignancies—B2M production may be increased. In the kidney, B2M is freely filtered at the glomerulus and is almost completely reabsorbed and catabolized by proximal tubular epithelial cells [204]. Therefore, reduced tubular reabsorption of B2M leads to enhanced urinary excretion and its utility as a sensitive marker of proximal tubular damage. Nevertheless, its presence in excess in the urine could be due to enhanced systemic secretion, in association with glomerular disorders, reducing its specificity [205].

Urinary B2M has been extensively evaluated as a surrogate marker of cisplatin-induced nephrotoxicity. Marginally increased levels of urinary B2M have been reported in patients undergoing cisplatin-based chemotherapy, very briefly after drug administration [189]. For instance, among a group of patients with ovarian carcinoma followed up for 24 weeks, there was an increase in urinary B2M excretion post-cisplatin administration, and this normalized after 2 weeks of each course of the drug administration pattern [206]. Additionally, among 57 patients administered cisplatin, urine B2M levels increased threefold on day 3 before other markers of tubular injury, such as KIM-1, TFF-3, or calbindin proteins, increased in urine or plasma [173]. Paradoxically, although there is an early surge in B2M levels, it has not been clearly shown that increases in B2M lead to or are associated with the development of overt acute kidney injury.

Its possible utility as a predictor of chronic cisplatin-induced renal dysfunction has also been evaluated. Glomerular filtration rate, measured by chromium-51-labeled EDTA clearance, was significantly reduced after repeated cycles of cisplatin treatment and remained depressed during long-term follow-up. Urinary excretion of B2M increased after each cycle of drug administration; however, correlations between peak B2M excretion and the long-term decline in GFR were poor, limiting its utility as an early predictor of chronic nephrotoxicity [188]. Other investigations similarly failed to demonstrate a reliable association between urinary B2M levels and long-term renal outcomes, and some cohorts showed no significant changes in B2M despite cisplatin exposure [207].

B2M has also been extensively evaluated in the context of cyclosporine-induced nephrotoxicity. In heart and bone marrow transplant recipients, elevated serum and urinary B2M levels have been associated with cyclosporine exposure and tubular injury [208]. Clinical studies have shown that urinary B2M increases before serum creatinine and declines with dose reduction, suggesting sensitivity to early tubular damage [209]. In renal transplant recipients, simultaneous measurement of serum and urinary B2M has been proposed as a tool to differentiate cyclosporine toxicity from acute rejection, with some studies reporting diagnostic accuracy approaching 90% [210]. However, other investigations have yielded conflicting results, demonstrating elevated urinary B2M in both rejection and toxicity, or no significant changes in tubular proteins despite clinical deterioration [211]. Consequently, no universally accepted B2M pattern reliably distinguishes between rejection and calcineurin inhibitor toxicity [212].

Elevated levels of B2M have additionally been demonstrated in the urine after aminoglycoside antibiotics, consistent with proximal tubule nephrotoxicity [213]. Experimental rat models found that B2M and albuminuria were more closely associated with histopathological changes after gentamicin exposure [214]. Clinical trials have reported similar elevations of B2M in the urine of both adults and children receiving aminoglycoside antibiotics, which precede elevations in serum creatinine by a few days [215]. Similar results have been found with other nephrotoxic medications, including ifosfamide and tenofovir, with excess B2M in urine reflecting proximal tubule dysfunction [216,217,218].

Altogether, urinary B2M levels have proved to be a sensitive marker of acute proximal tubule injury and often increase after exposure to nephrotoxic drugs like cisplatin, cyclosporine, aminoglycosides, and certain antiviral medications [193]. Nevertheless, its specificity and predictive performance as a renal diagnosis and GFR risk marker have remained poor, due to fluctuations in systemic production and the lack of consistent correlation between its levels and clinical cases of acute kidney injury or reduced GFR. It should thus be used as a marker in concert with other renal and injury type markers.

### 5.6. Clusterin

Clusterin is a highly glycosylated stress protein with a wide expression profile in tissues and cells, including the kidneys. It acts in a context-specific manner in cellular survival, apoptosis, and tissue remodeling processes [219]. In the human system, there are two main isoforms of the protein: secretory and cytoprotective, and nuclear and pro-apoptotic [220]. These isoforms make the protein involved in the regulation of the cell cycle and apoptosis. In the kidneys, it is involved in the aggregation and repair of damaged epithelial cells.

Clusterin is normally expressed at low levels in the kidney under physiological conditions [221]. On tubular injury, there is an immediate increase in the expression of clusterin messenger RNA and protein, especially in injured tubular epithelial cells. More importantly, unlike many plasma proteins, clusterin is not freely filtered from the blood in the glomerulus; accordingly, its presence in urine is a marker of local tubular production rather than glomerular disease [222].

In preclinical studies, urinary clusterin has been established as a biomarker for drug-induced nephrotoxicity [223]. In an extensive toxicology study involving more than 700 animals, urinary clusterin was shown to be more effective than the conventional biomarkers, blood urea nitrogen and serum creatinine, in assessing proximal tubular damage induced by drugs such as cisplatin, gentamicin, vancomycin, tacrolimus, puromycin, and doxorubicin [224]. Its limited sensitivity to glomerular damage, due to its non-expression in glomeruli, further supports its specificity for tubular damage. In multi-biomarker assessments, clusterin, KIM-1, and albumin ranked among the most sensitive for detecting urinary drug-induced tubular damage [225].

Clusterin has been shown to differentiate tubular from glomerular proteinuria in animal models of renal disease [226]. In diseases associated with tubular damage, such as bilateral renal ischemia and polycystic kidney disease, urinary clusterin levels increase in tandem with tubular injury. Conversely, models of focal segmental glomerulosclerosis result in heavy proteinuria and albuminuria without a concomitant increase in urinary clusterin, supporting its specificity for tubular epithelial injury [227].

Further studies have demonstrated that clusterin is a marker of injury across various tubular segments. In rat models exposed to cisplatin, gentamicin, and N-phenylanthranilic acid, urinary levels of clusterin correlated with injuries in the proximal tubule, distal tubule, and collecting duct, specifically during periods of renal epithelial regeneration [228]. Based on data from various studies, clusterin, along with KIM-1 and other biomarkers in urine, was identified as a valid additional parameter in preclinical toxicity studies by both the European Medicines Agency and the United States’ Food and Drug Administration [224].

Cisplatin-specific studies have further upheld the susceptibility of the urine clusterin. In animal models, urine clusterin was elevated following both low and high doses of cisplatin, prior to the manifestation of functional abnormalities [92]. Also, clusterin expression was elevated from the first day of cisplatin administration and correlated closely with histopathological manifestations of tubular damage. In several studies, urine clusterin and tissue KIM-1 have been identified as the earliest markers of cisplatin-induced nephrotoxicity [229,230]. Similar observations have been made regarding gentamicin-induced nephrotoxicity, in which urine clusterin persisted longer than NAG [231].

In brief, urinary clusterin is a sensitive, injury-specific marker of tubular epithelial injury, especially in nephrotoxicity induced by cisplatin, gentamicin, and other tubular toxins. This is because urinary clusterin is not subject to glomerular filtration, which helps increase specificity in interpreting test results and clearly distinguishes tubular injury from glomerular injury as a source of proteinuria. Notwithstanding the established view of urinary clusterin as a specific urinary marker of tubular epithelial injury, most available information has been obtained primarily through experimentation in laboratory animals and warrants clinical assessment of human drug-induced nephrotoxicity.

### 5.7. Trefoil Factor 3 (TFF3)

Trefoil factor 3 (TFF3) is a small peptide secreted in abundance by mucus-producing epithelial cells, especially in the gastrointestinal tract, where TFF3 has been implicated in the protection and repair of the epithelial layer [232]. TFF3 is expressed in kidney collecting duct epithelial cells; its function in renal tissue is not clearly understood [205].

Clinical investigations have shown that TFF3 levels are significantly elevated in urine and plasma in patients with CKD [233,234]. Baseline urinary TFF3 levels are significantly influenced by factors such as African origin, diabetes, and antihypertensive drug therapy, suggesting that TFF3 levels may serve as a marker of renal remodeling or repair in these patients [234]. A community-based study of 2948 participants from the Framingham Heart Study showed that urinary TFF3 levels, combined with other biomarkers, independently predicted both overall and kidney disease-related mortality [235]. This observation suggests that TFF3 levels might be used as a predictor of CKD progression, similar to albuminuria levels. Further validation of this hypothesis is needed.

Unlike the CKD-related increases, preclinical nephrotoxicity has more commonly resulted in decreased urinary TFF3. In one study employing a rat model of toxicants targeting various nephron compartments, including proximal tubular toxins (e.g., cisplatin, gentamicin), glomerular toxins, vascular toxins, and interstitial toxins, decreases in urinary TFF3 were observed concomitant with rising albuminuria and tubular injury [187]. Consistent with this latter study, treating rats with cisplatin (1.0 or 2.5 mg/kg) resulted in increased renal KIM-1 and clusterin expression within 5 days, with accompanying decreased urinary TFF3 levels and unchanged β2-microglobulin levels [236]. These data indicate the potential for loss or suppression of the TFF3 expression accompanying acute tubular epithelial injury in experimental models.

Human information on TFF3 expression in relation to nephrotoxicity induced by medications is sparse [237]. In one clinical study of 57 patients with solid malignancies treated with cisplatin on an outpatient basis, urinary TFF3 levels increased twofold by day 10 of therapy, accompanied by concomitant elevations in KIM-1. This is inconsistent with the reductions observed in animal models, and there is no clear understanding of interspecies differences at the biological level [238].

TFF3 thus appears to be an indicator of epithelial remodeling and disease progression in chronic kidney disease. However, its use as a marker of acute drug-induced nephrotoxicity is unclear [237]. Animal models have shown reduced TFF3 secretion following tubular injury. However, there is scant evidence of increased TFF3 secretion in human models of cisplatin exposure. These seemingly conflicting observations suggest a need for human investigation into its use as a marker of nephrotoxicity.

### 5.8. Netrin

Netrins are a group of secreted guidance cues with a laminin-like topological structure and domain arrangement. They are classified as laminin-related axon guidance cues and are encoded by separate genes, including netrin-1, netrin-3, and netrin-4 [239]. Netrins mainly signal through Deleted in Colorectal Cancer (DCC) and Unc-5 receptors to modulate cell migration, adhesion, and survival. Netrins, known to act as guidance cues in neural development, are multifunctional and participate in other physiological processes, such as organogenesis, angiogenesis, and immune responses [240].

In addition to their roles in development, netrins have been reported to have significant anti-inflammatory properties, inhibiting leukocyte chemotaxis and stimulating endothelial cell migration. Among the netrins, netrin-1 was found to inhibit leukocyte infiltration and enhance vascular repair. In the kidney, netrin-1 is expressed at low levels under normal conditions but is rapidly induced upon injury.

Experimental models of ischemic acute kidney injury (AKI) have shown that netrin-1 is an early and transient urinary biomarker of renal tubular injury. Urinary netrin-1 concentrations have been shown to increase significantly after three hours of injury and return towards baseline levels at 72 h in mouse ischemia/reperfusion models, while plasma creatinine concentrations increase significantly after 24 h of injury. These observations establish netrin-1 as an early biomarker of renal injury, given its transit nature and its low baseline levels in normal control animals [241].

There are limited data from human studies to validate these experimental observations. Increased netrin-1 excretion has been documented to be associated with AKI of various etiologies, including ischemic, septic, radiocontrast-induced, drug-induced, and post-transplant AKI. In a comparative cohort study, the netrin-1-to-creatinine ratio was significantly higher in patients with AKI than in healthy individuals, suggesting responsiveness to incipient renal insults of various etiologies.

Despite these encouraging findings, the current state of knowledge regarding the specific role of netrin-1 in DR nephrotoxicity is very limited. The available studies address IK/AKI in a very broad manner. Consequently, systematic assessments of specific nephrotoxic exposures, dose–response correlations, and long-term follow-up are not available. At the same time, netrin-1 shows strong potential as an early, inflammation-linked marker of tubular injury; further mechanistic and clinical studies are required to establish its diagnostic and prognostic utility in drug-induced kidney injury.

### 5.9. Interleukin-18 (IL-18)

Interleukin-18 (IL-18) is a pro-inflammatory cytokine that plays an important role in both innate and adaptive immune responses [242]. It is produced predominantly by activated macrophages but is also expressed by mononuclear cells, renal and intestinal epithelial cells, keratinocytes, dendritic cells, and osteoblasts [243]. Through its ability to stimulate interferon-γ production and amplify inflammatory signaling, IL-18 contributes to host defense against infection and malignancy, while also participating in tissue injury under pathological conditions.

IL-18 has been extensively studied in the context of ischemic AKI. In experimental models, renal and urinary IL-18 levels rise rapidly following ischemia–reperfusion injury [244]. Neutralization of IL-18 before ischemic insult, genetic overexpression of IL-18-binding protein, or pharmacologic inhibition of IL-18 activity have been shown to attenuate both functional impairment and histological damage in murine models, supporting a pathogenic role in ischemic AKI [245,246,247]. Translational studies from the TRIBE-AKI consortium further demonstrated that urinary IL-18 levels increase within six hours of ischemic injury—at least one day before elevations in serum creatinine—and are predictive of both short- and long-term renal outcomes [248].

Based on these findings, IL-18 has also been investigated as a biomarker of drug-induced nephrotoxicity. In animal models, a high dose of cisplatin was associated with a marked increase in renal and urinary IL-18 levels, as well as in IL-1β and IL-6 [249]. This was also observed in a murine study, in which a large increase in TNF-α, IL-18, and KIM-1 levels was noted in conjunction with cisplatin [250]. Notwithstanding, neutralization of IL-18 did not protect against cisplatin-mediated AKI [249]. This is important because it suggests that IL-18 is a biomarker rather than a mediator in these instances.

Clinical data on human IL-18 expression in drug-related kidney injury are sparse but indicate a possible role as an adjunct biomarker [250]. In one study of a limited number of patients treated with cisplatin-based chemotherapy, urine levels of IL-18, leucine aminopeptidase, NGAL, cystatin C, and L-FABP were upregulated three hours after drug exposure, approximately 72 h before an increase in serum creatinine concentrations [251]. Additional pediatric studies have reported early increases in urinary IL-18 and NGAL in children treated with ifosfamide, cisplatin, or carboplatin, and these elevations were associated with both acute and chronic nephropathy [252]. Urinary IL-18 has also been linked to contrast-induced nephropathy and adverse cardiovascular outcomes following coronary angiography.

In summary, urinary IL-18 is a well-validated early biomarker of ischemic AKI and reflects inflammatory activation within the injured kidney. Emerging evidence suggests that IL-18 may also serve as an early indicator of nephrotoxin-induced renal injury; however, available clinical studies are limited by small sample sizes and heterogeneous patient populations. Larger, well-controlled studies are required to establish the diagnostic specificity, prognostic value, and clinical applicability of IL-18 in drug-induced nephrotoxicity.

### 5.10. Cystatin C

Cystatin C is a low-molecular-weight cysteine protease inhibitor that is constitutively produced by all nucleated cells and released into the circulation at a relatively constant rate [253]. Because it is almost completely reabsorbed and catabolized by proximal tubular epithelial cells, cystatin C has emerged as a useful marker of GFR that is less influenced by age, sex, muscle mass, or diet than serum creatinine.

Several studies have demonstrated that serum cystatin C provides a more accurate estimate of GFR than creatinine when compared with reference clearance methods. In ^51^Cr-EDTA clearance studies, serum cystatin C showed superior correlation with measured GFR relative to serum creatinine [254]. Cystatin C-based estimating equations perform comparably to the Modification of Diet in Renal Disease (MDRD) formula and, in some settings, offer improved precision [255]. In patients undergoing cardiac catheterization, serum cystatin C exhibited a stronger association with iopromide clearance than serum creatinine or creatinine-based equations [256]. Similar findings have been reported in comparisons of Tc-99m DTPA clearance, in which cystatin C correlated more closely with measured GFR than β2-microglobulin or creatinine [257].

In addition to its role as a functional marker, urinary cystatin C has been proposed as an indicator of proximal tubular dysfunction, since intact tubules normally reabsorb filtered cystatin C. Accordingly, the appearance of cystatin C in urine reflects impaired tubular uptake and cellular injury. Urinary cystatin C has been shown to increase early in ischemic AKI and in some forms of renal toxicity [248,258]. However, the outcome, particularly with cisplatin in drug-induced nephrotoxicity, has been inconsistent.

Cisplatin-associated nephrotoxicity is one of the most extensively studied contexts for cystatin C, with variable results. Several clinical studies reported predictable increases in serum cystatin C following cisplatin exposure, without significant changes in serum creatinine [259]. In one cohort of 41 patients receiving cisplatin, serum cystatin C increased by approximately 21%, while inulin clearance was reduced by 23%, with little change in creatinine [260]. In pediatric oncology populations, equations that include cystatin C are useful for estimating GFR after nephrotoxic therapy [261]. By contrast, other studies have found that estimates of GFR derived from cystatin C are less reliable than those from renal scintigraphy or creatinine-based measures [262,263].

The performance of urinary cystatin C as a marker of cisplatin nephrotoxicity has been particularly inconsistent. Some studies reported no significant increase or no discrimination between AKI and non-AKI patients shortly after cisplatin administration, whereas others observed delayed elevations several days after treatment [45]. A meta-analysis of pediatric cancer patients suggested that cystatin C outperformed creatinine in detecting glomerular dysfunction; however, this advantage was not consistent across studies [264].

In contrast to cisplatin, serum cystatin C has shown more consistent performance in nephrotoxicity induced by other agents. In patients treated with amphotericin B, cystatin C-based GFR estimates correlated well with Cockcroft–Gault calculations early during therapy [265]. In radiocontrast-induced nephropathy, serum cystatin C increased concurrently with or earlier than serum creatinine [266,267]. Among pediatric leukemia patients. receiving high-dose methotrexate, serum cystatin C was a sensitive marker of renal function during and after treatment [268]. Similarly, in patients with cystic fibrosis treated with amikacin, serum cystatin C was a superior predictor of GFR and drug clearance compared with creatinine [269]. Experimental and clinical investigations have also demonstrated the value of serum cystatin C in assessing nephrotoxicity induced by colistin, aminoglycosides, and vancomycin, often performing better than serum creatinine in these settings [270,271,272].

In brief, serum cystatin C is a reliable surrogate marker for AKI diagnosis and nephrotoxicity evaluation across various drug classes, including aminoglycosides, amphotericin B, contrast agents, methotrexate, and vancomycin. The reliability in cisplatin-induced nephrotoxicity, especially in urinary assessment, is erratic. Due to its sensitivity and reduced reliance on non-renal variables, serum cystatin C has been used in regulatory and drug development settings as an adjunctive surrogate marker for renal safety evaluation [224]. Limitations include inter-assay variability, higher cost, and inconsistent utility in urinary measurements.

A detailed summary Table 2 has been provided to highlight the essential indicators of drug-induced nephrotoxicity, including their origins, affected organs, underlying mechanisms, and clinical significance. It provides an integrated overview to support early diagnosis, mechanistic understanding, and optimized clinical monitoring of renal toxicity.

### 5.11. Emerging Biomarkers of Drug-Induced Nephrotoxicity

Beyond the usual suspects like serum creatinine and blood urea nitrogen (which often lag behind actual kidney damage), a wave of newer biomarkers has really stepped up, helping spot drug-induced nephrotoxicity earlier and giving clearer clues about what’s going wrong inside the kidney. These are not just lab curiosities; they are starting to change how we think about catching trouble before it becomes obvious [273].

Two standouts are kidney injury molecule-1 (KIM-1) and neutrophil gelatinase-associated lipocalin (NGAL). Both tend to spike much sooner than creatinine and are directly tied to injury in tubular epithelial cells, think proximal tubule damage from drugs like cisplatin, gentamicin, or vancomycin. KIM-1, a transmembrane protein that is barely expressed in healthy kidneys but shoots up after injury, is especially good at flagging proximal tubular harm. NGAL, released from damaged tubules (and sometimes neutrophils), often rises within hours of an insult [274].

Then there are supporting players like β2-microglobulin, clusterin, trefoil factor 3 (TFF3), netrin-1, liver-type fatty acid-binding protein (L-FABP), and signs of albuminuria or proteinuria. These add layers: β2-microglobulin and cystatin C point to proximal tubule dysfunction (since they are normally reabsorbed there), clusterin flags regeneration or broader injury, TFF3 drops in response to collecting duct stress, and L-FABP reflects hypoxic or oxidative stress in the tubules. Albuminuria/proteinuria can suggest glomerular involvement or tubular overflow [275].

What makes these really exciting is how they bridge the gap between clinical signs and the underlying biology. KIM-1 and NGAL often scream “tubular injury” early, while panels mixing functional markers (like cystatin C) with mechanistic ones (oxidative stress, inflammation, mitochondrial trouble, or cell death pathways) paint a fuller picture of the damage. No single biomarker nails everything, but combinations catch the complexity better than going solo [273,276].

Looking ahead, as we refine antioxidant and other protective strategies (like the ones we talked about before), these biomarkers could help spot who’s at highest risk, track if a therapy is actually working, and guide timely tweaks before full-blown kidney function tanks. That is significant for safer drug use, especially with chemo agents, antibiotics, or contrast dyes, since waiting for creatinine to rise is often too late.

Bottom line: Integrating these sensitive, mechanism-linked biomarkers is a game changer for diagnosing and managing drug-induced nephrotoxicity more proactively and precisely.

**Figure 4:**
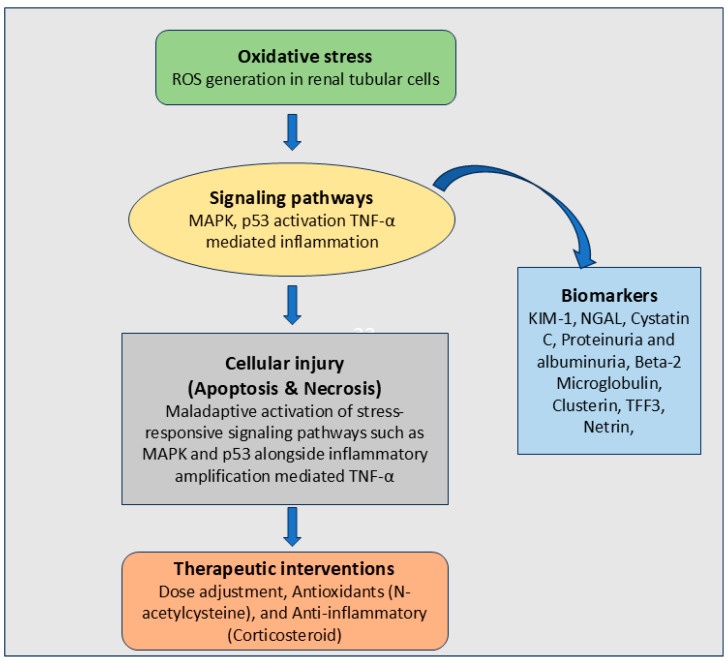
Integrated overview of oxidative stress, downstream signaling, biomarkers, and therapeutic interventions in drug-induced nephrotoxicity.

To better integrate the mechanistic, diagnostic, and translational aspects of drug-induced nephrotoxicity, Figure 4 summarizes the relationship among oxidative stress, downstream injury pathways, renal damage biomarkers, and potential therapeutic interventions. This framework highlights how excessive ROS generation and redox imbalance contribute to inflammation, apoptosis, and tubular injury, while also emphasizing the potential role of emerging biomarkers and antioxidant-based strategies in early detection and nephroprotection.

Oxidative stress induced by excessive ROS generation in renal tubular cells activates MAPK-, p53-, and TNF-α-related signaling pathways, leading to inflammation, apoptosis, necrosis, and progressive cellular injury. These events are associated with changes in biomarkers of renal damage, including KIM-1, NGAL, cystatin C, proteinuria, albuminuria, beta-2 microglobulin, clusterin, TFF3, and netrin. Therapeutic approaches aimed at limiting nephrotoxicity include dose adjustment, antioxidants such as N-acetylcysteine, and anti-inflammatory agents such as corticosteroids. Abbreviations: ROS, reactive oxygen species; MAPK, mitogen-activated protein kinase; TNF-α, tumor necrosis factor alpha; KIM-1, kidney injury molecule-1; NGAL, neutrophil gelatinase-associated lipocalin; TFF3, trefoil factor 3.

## 6. Treatment for a Drug Causing Nephrotoxicity

Patients who suffer acute drug nephrotoxicity may require hospitalization for care. The immediate goal of therapy is to restore renal perfusion to adequately supply renal tissue with oxygen and nutrients, facilitating recovery. Failure to seek timely and appropriate medical attention may lead to irreversible renal damage and even death. This is especially true in those with ESRD [277].

Hypovolemia is a significant and potentially correctable risk factor for drug-induced renal toxicity. Thus, volume and hemodynamic stabilization are key parts of management. Fluid management to correct should be based on the principle of supporting homeostasis. Patients with intravascular volume depletion will most likely require isotonic fluids, such as normal saline, rather than hypertonic fluids or colloids, such as starches or albumin [278]. For patients with vasodilatory or vasomotor shock, vasopressor support with the agent of choice (e.g., norepinephrine) may be needed to stabilize mean arterial pressure [279].

It should further include the following because of their potential impact on the renal injury caused by the drug. It should take into account all the patient’s other factors, because treating one disease should not affect the others. It should not follow a standard treatment procedure.

Once nephrotoxicity is suspected or documented because of medications, it is recommended to discontinue the causative drug, if clinically possible. This is especially helpful in reducing the extent of nephrotoxicity in severely nephrotoxic drugs, such as cisplatin, amphotericin B, and aminoglycosides, where early drug withdrawal can prevent and even reverse severe renal impairment [280]. For severe and progressive renal impairment, the need for renal replacement therapy, such as initiating dialysis, may be invoked. This, in turn, is based on clinical and disease-associated KDIGO guidelines [281].

Prevention measures for nephrotoxicity associated with drug use remain few in number but remain crucial, especially in high-risk patient groups like the elderly population, those presenting with existing renal disorders, and those on multiple drug regimens. Early recognition of susceptible patient groups, dosage titration, and preventive supportive care remain key components in prevention and patient care strategies [20]. This must be carried out alongside the detection of renal injury biomarkers to enable preventive measures to be taken before permanent renal injury. Customized and individualized drug therapy based on patient medication history and therapeutic drug monitoring may also circumvent the onset of renal adverse events.

Clinically, prevention of drug-induced nephrotoxicity should emphasize patient-specific risk stratification, renal function-guided dose adjustment, avoidance of overlapping nephrotoxins, and standardized hydration protocols to limit renal drug accumulation and oxidative injury [85,86,89]. Recent clinical trials and cohort studies further support the use of biomarker-guided monitoring to enable early intervention before irreversible damage occurs [130,132,138]. Adjunctive strategies targeting oxidative stress and mitochondrial dysfunction have also shown renoprotective potential in selected clinical settings [39,83,282].

### Antioxidant-Based Strategies to Prevent or Mitigate Drug-Induced Nephrotoxicity

Oxidative stress is a major driver behind drug-induced kidney damage, which is why it has become such a promising target for ways to protect the kidneys. Many common medications can ramp up the production of reactive oxygen species (ROS), throw off mitochondrial balance, kickstart lipid peroxidation, deplete the body’s natural antioxidants (like glutathione), and fire up inflammation via redox-sensitive signaling. All of this can harm kidney tubules, disrupt the lining of renal blood vessels, and slowly worsen overall kidney function [12,137].

Scientists have looked at a bunch of antioxidant strategies as add-on treatments in lab and animal studies of drug-related kidney toxicity. These include compounds that mop up ROS directly, agents that help rebuild glutathione, agents that shield mitochondria, and others that rev up the body’s built-in antioxidant machinery, especially the Nrf2 pathway, which flips on genes for detox and antioxidant enzymes [283,284].

N-acetylcysteine (NAC) is probably the most researched of the bunch. It boosts glutathione levels, reduces oxidative damage, and dials back inflammation. In animal models, NAC has protected against cisplatin-induced kidney toxicity, often by blocking C5a receptor activation, and shown promise against vancomycin and other drugs. However, human trial results (for cisplatin, colistin, etc.) can be inconsistent depending on timing, dose, and patient specifics [285,286].

Plant-based polyphenols and flavonoids have also turned heads. Curcumin (the active part of turmeric) eases oxidative stress, helps keep mitochondria healthy, and prevents cell death in models of cisplatin and similar kidney injuries. It often works by activating Nrf2/Keap1 signaling, reducing inflammation, and improving mitochondrial function through pathways such as AMPK/PGC-1α/Sirt3 [287]. Resveratrol (from grapes, red wine, and berries) guards against oxidative damage, inflammation, and programmed cell death in lab models of cisplatin toxicity, sometimes by reducing the amount of the drug that accumulates in the kidney [287,288]. Quercetin does something similar, cutting oxidative and nitrosative stress while improving kidney markers in various toxicity models [288,289].

Then there are other compounds like melatonin, coenzyme Q10, alpha-lipoic acid, vitamins C and E, selenium, and even mitochondria-targeted antioxidants. How well they work depends on the drug and setup—alpha-lipoic acid, for example, triggers protective responses across different kidney injury types; melatonin frequently matches or beats others at restoring glutathione and limiting damage; and combos (like melatonin plus alpha-lipoic acid) can give extra benefit [290].

It is important to realize that antioxidant protection is not just about blanket ROS cleanup. The best results usually come from targeting specific trouble spots such as mitochondrial dysfunction, ferroptosis (an iron-linked form of cell death), inflammation, ER stress, or certain cell-death pathways in kidney cells. Things that strengthen the body’s own defenses, maintain mitochondrial energy, or prevent lipid peroxidation often stand out. Combining antioxidants can be especially useful when oxidative stress interacts with inflammation, vascular issues, or immune responses [12].

Bringing these ideas into actual patient treatment is not straightforward. Success often rides on when you give the antioxidant, the right dose and route, which exact drug is causing the trouble, and patient factors like starting kidney function, diabetes, or chronic inflammation. Above all, any protective add-on must avoid weakening the main job of drugs like chemotherapy agents or antibiotics.

In the future, the most useful advances will probably come from antioxidants designed for precise redox mechanisms, using biomarkers to pick the right patients, and smarter trial designs that connect solid lab/animal data to what really happens in clinics.

All in all, antioxidant strategies make good biological and clinical sense for preventing or mitigating drug-induced kidney toxicity. Getting a better grip on how redox signals unfold in the kidney, identifying who’s most vulnerable, and nailing the best timing will be key to turning these into everyday tools for kidney protection.

A summary of treatment modalities for addressing renal toxicity issues is presented below (Table 3).

## 7. Strategies for Drug-Induced Nephrotoxicity Study: Preclinical Prediction of Nephrotoxicity

Identification of nephrotoxic risk early on is a key point in the development of a drug, and failure to recognize the presence of renal toxicity within preclinical studies has contributed heavily to the attrition rate of program failure in the latter phases of development. To reduce the risk of nephrotoxicity, predicting nephrotoxicity caused by a drug has become a standard part of a drug’s safety profile during preclinical development. Although improved methodologies for nephrotoxicity screening exist, 90% of lead compounds have been eliminated from the pharmaceutical development process due to a variety of factors, including renal toxicity [291].

Owing to the kidney’s pre-eminent role in the elimination, concentration, and biotransformation of medications, the kidney is highly sensitive to toxicity and is the primary focus of preclinical studies. Modern nephrotoxicity testing involves the application of both in vitro and in vivo models. The former utilizes conventional two-dimensional human renal cell cultures, sophisticated three-dimensional (3D) cell models, and microfluidic “kidney on a chip” devices, which facilitate the manipulation and simulation of tubular transport and cellular interaction. These models deliver mechanistic data on cellular toxicity, substrate uptake, oxidative damage, and inflammation, which currently depend less on animal models.

In vivo animal models remain indispensable for assessing integrated renal responses, including hemodynamic changes, immune activation, and multi-organ interactions, which are poorly recapitulated in vitro. When coupled with sensitive injury biomarkers, histopathology, and functional readouts, animal studies allow for the detection of early renal injury and the characterization of dose–response relationships. Importantly, incorporating translational biomarkers into preclinical studies enhances alignment with clinical endpoints and improves the prediction of human nephrotoxicity.

In general, integrated preclinical platforms for early-stage nephrotoxicity evaluation support informed compound selection and dose optimization, as well as risk mitigation before clinical testing. These strategies further reduce costs and the ethical burden while increasing the likelihood of advancing safer, more effective therapeutics into clinical trials.

### 7.1. Two-Dimensional Cell Culture Models

In light of this reality, drug-induced nephrotoxicity linked to newly approved therapeutics continues to be a leading clinical concern that is often poorly recognized in early drug development. In efforts to surmount this hurdle, serious attempts have focused on studying renal toxicity using in vitro screening platforms, the majority of which are 2D cell culture models. Primary renal cells and immortalized cell lines are the most commonly used cell types because they are readily available, amenable to experimentation, and suitable for mechanistic studies of nephrotoxicity [291].

Two-dimensional monolayers offer several unique benefits for large-scale compound screening, enabling rapid cytotoxicity evaluation at a reasonable cost and with fewer ethical issues than animal models [292]. The model has been widely used to study cytotoxicity, oxidative stress, and inflammation induced by compounds under controlled conditions. Numerous assays have been developed and employed for assessing various facets of nephrotoxicity in a 2D model, such as evaluating cytotoxicity induced by compounds using various assays such as Alamar Blue, CellTiter-Blue, and DNA content assays, as well as using membrane integrity and cytotoxicity assays such as dye exclusion and incorporation assays using various fluorescent agents such as Hoechst, calcein-AM, and so on.

Despite their utility, 2D cell culture models have important limitations. The kidney is a highly complex organ composed of multiple specialized cell types, including proximal and distal tubular epithelial cells, collecting duct cells, cells of the loop of Henle, endothelial cells, mesangial cells, and podocytes, each contributing uniquely to renal function and drug handling. As such, no singular cell line can capture the structural, functional, and metabolic breadth of the kidney. In addition, commonly utilized renal epithelial cell lines often lack critical drug transporters and metabolic enzymes, particularly those characteristics of proximal tubular cells, which are centrally involved in drug uptake and nephrotoxicity [293].

As a result, using a single cell type in 2D models could lead to under- or erroneous assessments of toxicity. To better translate to the human system and to more effectively analyze the region-specific mechanism of toxicity, studies on nephrotoxicity could be aided by using several varieties of renal cells derived from different nephron segments. Even so, the weaknesses identified in 2D cell culture models make the application of more advanced models necessary.

### 7.2. Three-Dimensional Organoid Models

Although traditional two-dimensional cell culture systems have contributed significantly to nephrotoxicity screening, they lack the key architectural and functional features of the kidney, including a native extracellular matrix, multicellular organization, and vascular-like structures. These can limit reproducibility and compromise the predictive accuracy of toxicity assessments. To overcome such constraints, greater emphasis has been placed on three-dimensional culture systems in which renal cells are embedded within extracellular matrix-based scaffolds that better recapitulate tissue-level organization [294].

Three-dimensional kidney models provide a more physiological microenvironment, in which cells can form spatial relationships, exhibit polarity, and display differentiation patterns similar to those observed in living organisms [295]. This, in turn, increases the sensitivity and predictive value of 3D cell cultures towards human drug responses, especially to those with mild and slow nephrotoxicity [293]. Among the models, cell organoids resembling nephrons and derived from human induced pluripotent stem cells represent an efficient system for nephrotoxicity assessment.

hiPSC-derived renal organoids comprise various components of the nephron, including proximal and distal tubules, glomerular rudiments, and regions resembling the loop of Henle, thus reflecting the complexity that cannot be provided by conventional 2D platforms [294]. Comparative analyses have revealed that organoids are more sensitive to nephrotoxic compounds and more predictive than conventional 2D cell cultures [296]. Notably, organoids offer the advantage of being amenable to chronic nephrotoxicity experiments, which would be impractical in conventional 2D models due to their short-term nature [297].

Recent progress has also improved 3D nephrotoxicity assay platforms by integrating microfluidics, leading to the development of the kidney-on-a-chip [298]. The platform is designed to host multiple types of renal cells in three dimensions, with regulated fluid dynamics, to simulate physiological forces, such as shear stress, solute transport, and drug concentration dynamics [299]. The use of microfluidics technology in the kid-on-a-chip has eliminated many challenges associated with three-dimensional cultures.

### 7.3. In Vivo Preclinical Animal Models

Despite their usefulness for gaining mechanistic insights, in vitro models are not adequate for the full understanding of nephrotoxicity induced by pharmaceuticals [300]. This aspect of kidney injury caused by pharmaceuticals indicates involvement of ADME properties, such as protein binding, and of mechanisms, such as immune system functions. Hence, small-animal models are critical for nephrotoxicity testing in biological systems [293].

The use of rodent models, especially mice and rats, has been favored because of their relevance to human physiology and their ability to be genetically modified and replicated. Acute kidney injuries can be consistently and faithfully replicated using known nephrotoxic compounds. Cisplatin, for instance, has been used to dose induce injuries to the proximal tubules of the kidneys, with cisplatin showing a predilection to accumulate within these target proximal tubular epithelial cells [301,302]. Other compounds, including aminoglycoside antibiotics, have been widely used to replicate acute injuries to the proximal tubules of the kidneys, reflecting human pathologies [192].

In vivo models permit a thorough evaluation of renal function, histopathology, biomarker kinetics, and systemic responses, such as inflammatory reactions and vascular alterations, that cannot be properly captured in an in vitro study. In vivo models also permit the study of pharmacokinetic and pharmacodynamic relationships, dosing, gender- and age-related susceptibility, and the presence of comorbid conditions such as diabetes or pre-existing renal disease. Moreover, animal studies enable validation of novel biomarkers and therapeutic approaches in a controlled research setting.

Animal models, with their translational value, raise several difficult ethical and regulatory principles that need consideration; this includes adherence to the principles of the 3Rs: Replacement, Reduction, and Refinement. Animal use must therefore be minimized while maximizing scientific rigor in the design of experiments and strategically combining animal studies with advanced in vitro systems to achieve predictive accuracy with reduced unnecessary experimentation.

### 7.4. Clinical Monitor of Nephrotoxicity

Close clinical monitoring is, therefore, crucial for the early detection and prevention of drug-induced kidney toxicity. Close monitoring would begin with identifying susceptible patient populations [303]. These would include the elderly (>60 years of age, infants, and patients already vulnerable due to underlying kidney problems, as well as patients receiving higher drug combinations. In susceptible patients, even slight damage can be sufficient to cause severe kidney problems [304].

However, once risk has been identified, it becomes important to focus on continuous monitoring of kidney function during drug treatment. The traditional functional measures of kidney function, such as serum creatinine and GFR calculations, remain in vogue but can and should be supplemented by new kidney injury biomarkers that allow earlier identification of subtle kidney injury, even before it occurs. Does individualization in drug treatment become important in view of significant variations in susceptibility to kidney injury? For instance, aminoglycoside antibiotics can be administered to optimize dosing and minimize potential kidney injury.

Mechanistic insights into how the kidneys manage drugs have unlocked new routes to counter toxicity. Organic cation transporters 2 (OCT2) are known to mediate cisplatin transport into proximal tubular epithelial cells, and pharmacologic and genetically mediated modulation of OCT2 has been shown to decrease cisplatin accumulation and toxicity in preclinical models [305]. Furthermore, additional approaches aimed at addressing the inflammatory and apoptotic components of damage have been shown to be renoprotective in the setting of cisplatin exposure, and it would seem that combination therapy may allow for sparing of kidney function without compromising efficacy against cancer [306].

Pharmacogenomics and pharmacogenetics have moved forward to provide further evidence for the use of personalized strategies for the prevention and monitoring of nephrotoxicity. Genetic differences in metabolizing enzymes, transporters, and inflammatory mediators affect the kidney’s susceptibility to toxicity. The use of genomic information in Phase I-III clinical trials enables stratification and the tailoring of dosing regimens, thereby optimizing the effectiveness of therapy and safety [307].

Personalized medicine has the promise of providing the greatest benefit with the least nephrotoxicity (Table 4).

## 8. Conclusions with Future Perspectives

Oxidative stress has recently emerged as a key unifying mechanism in drug-induced nephrotoxicity. Regardless of drug class—whether chemotherapeutic, immunosuppressive, antimicrobial, or contrast agents—excessive ROS production disrupts redox balance in the kidney’s metabolically active tubular epithelium, triggering stress responses, amplifying inflammation, damaging mitochondria, and ultimately activating cell death programs. The high energetic workload, poor regenerative capacity, and a highly refined redox-sensitive antioxidant system in the kidney make it even more susceptible to changes in redox state.

Significantly, an increasingly evident finding suggests that nephrotoxicity can be caused by a failure of protective mechanisms to suppress oxidative and inflammatory damage, and not only by cellular toxicity. An imbalance of protective mechanisms, inadequate activation of cytoprotective signaling, and inflammation can be key determinants of the reversibility of renal damage or progression to chronic renal damage.

In translational terms, these observations point to several important considerations for the future. Firstly, there is a need to identify redox vulnerabilities in a kidney- and cell-type-specific manner, particularly within specific nephron segments, to better understand risk. Secondly, a merging of oxidative stress response biomarkers with functional and injury biomarkers will hopefully provide an earlier indication of nephrotoxicity. Lastly, further research into redox-targeting therapy in the form of mitochondrial-targeting agents, boosters of the cellular redox defense, or modulators of pathological inflammation would likely provide an important means of modulating renal injury without affecting therapeutic outcomes.

Experimental models should be selected based on drug-specific mechanisms, with proximal tubular models for cisplatin and aminoglycosides, vascular and endothelial models for calcineurin inhibitors, and immune-mediated systems for β-lactams and biologics. In clinical settings, integrating multi-biomarker panels with genetic and metabolic profiling may enable early risk stratification and individualized therapy.

Furthermore, therapeutic strategies targeting mitochondrial protection, enhancing endogenous antioxidant defenses, and modulating pathological inflammation should be systematically evaluated in preclinical and clinical studies. The development of precision medicine approaches combining mechanistic biomarkers, pharmacokinetic modeling, and patient-specific risk factors will be essential for optimizing treatment efficacy while minimizing renal toxicity. Collectively, these efforts will facilitate the translation of redox biology into clinically actionable interventions for improved renal safety.

Finally, advances in pre-clinical modeling and precision medicine also provide opportunities to translate redox biology to clinically actionable approaches. A comprehensive translational platform that integrates understanding of oxidative stress and employs precision medicine approaches will be necessary to ensure enhanced renal safety and maintenance of kidney function in patients undergoing nephrotoxic therapies.

## Figures and Tables

**Table 1:** Drug classes associated with nephrotoxicity, clinical manifestations, and underlying pathophysiologic mechanisms.

Drugs Class	Drugs Name Active Ingredients (Generic Name)	Clinical Symptoms Primary Sites of Renal Injury	The Pathophysiological Process That Governs Renal Damage	References
Analgesics	Aspirin, Acetaminophen, Nonsteroidal anti-inflammatory drugs (NSAIDs)	Glomerular disease, Hemodynamic alteration, Acute allergic interstitial nephritis	Changing intraglomerular hemodynamics, acute interstitial nephritis, glomerulonephritis, prolonged interstitial nephritis	[78]
Aminoglycosides	Gentamicin, Tobramycin, Amikacin	Acute tubular necrosis and reduced GFR	Proximal tubular accumulation, acute interstitial nephritis, lysosomal rupture, oxidative stress, mitochondrial dysfunction, tubular epithelial cell apoptosis, and necrosis	[79]
β-lactam antibiotics	Cephalosporins, Penicillin’s	Acute interstitial nephritis, mild proteinuria	Inflammatory infiltration of the interstitium, tubular and glomerular inflammation, Immune-mediated hypersensitivityreactions,	[79]
Immunosuppressive agents	Tacrolimus, Cyclosporine	Hemodynamic alteration, Distal tubule injury, chronic kidney disease	Prolonged intraglomerular hemodynamics, oxidative stress, tubular atrophy, and interstitial fibrosis	[80,81]
Antiretrovirals	Adefovir, Cidofovir, Tenofovir, Indinavir	Proximal Tubule injury, Fanconi syndrome, reduced GFR	Tubular cell toxicity, crystal precipitation (Indinavir), Mitochondrial toxicity, proximal tubular transporter dysfunction, oxidative tubular stress	[82]
Chemotherapeutics	Cisplatin, Carboplatin, Ifosfamide, Methotrexate, Doxorubicin	Acute tubular necrosis, hemodynamic changes, Mild acute tubular injury, reduced GFR, Proximal tubular dysfunction, Fanconi-like syndrome Acute kidney injury, crystal nephropathy, Glomerular and tubular injury	Tubular cell toxicity, Prolonged interstitial nephritis, Tubular precipitation, oxidative tubular stress, and inflammatory injury, ROS-mediated mitochondrial damage, endothelial dysfunction	[83,84,85]
Radio Contrast Agents	Iodinated contrast media	Hemodynamic alteration	Tubular cell toxicity	[83]

**Table 2:** Summary on biomarkers for drug-induced nephrotoxicity.

Biomarker	Source	Primary Site of Injury	Mechanism Reflected	Drugs	Clinical Utility	Limitations
Serum creatinine	Blood	Global kidney	Reduced GFR	All nephrotoxic drugs	Routine renal monitoring	Late marker, low sensitivity
BUN	Blood	Global kidney	Impaired excretion	All nephrotoxic drugs	Supportive indicator	Non-specific
GFR (Inulin, DTPA, Iothalamate)	Blood /Urine	Global kidney	Filtration capacity	All nephrotoxic drugs	Accurate function assessment	Invasive, costly
KIM-1	Urine	Proximal tubule	Tubular Apoptosis /necrosis	Cisplatin, Gentamicin, Cyclosporine	Early tubular injury detection	Time-dependent variability
Albuminurea/Proteinuria	Urine	Glomerulus/Tubule	Barrier dysfunction	Cisplatin, Cyclosporine	Glomerular involvement	Poor AKI prediction
NGAL	Urine/Blood	Tubule	Oxidative stress, inflammation	Cisplatin, Aminoglycosides, Amphotericin B	Early AKI marker	Variable specificity
NAG	Urine	Proximal tubule	Lysosomal damage	Contrast media, Aminoglycosides	Tubular injury indicator	Low specificity
β2- Microglobulin	Urine /Blood	Proximal tubule	Reabsorption defect	Cisplatin, Cyclosporine, Tenofovir	Early tubular dysfunction	Affected by systemic levels
Clusterin	Urine	Tubule	Cellular stress, repair	Cisplatin, Gentamicin, Vancomycin	Sensitive tubular marker	Limited human data
TFF3	Urine /Plasma	Collecting duct	Epithelial remodeling	Cisplatin	CKD progression marker	Inconsistent in AKI
Netrin-1	Urine	Tubule	Inflammation, repair	Cisplatin, Contrast agents	Early injury marker	Limited clinical validation
IL-18	Urine	Tubulointerstitium	Inflammatory injury	Cisplatin, Ifosfamide	Early AKI indicator	Not disease- specific
Cystatin C	Blood /Urine	Glomerulus/Tubule	GFR reduction, uptake failure	Aminoglycosides, Methotrexate, Contrast	Sensitive GFR marker	Variable in cisplatin

**Table 3:** Treatment Modalities for Drug-Induced Nephrotoxicity.

Drugs/Agents	Nephrotoxic Effect	Associated Conditions	Management
EGFR inhibitors (Cetuximab, Panitumumab)	Inhibition of EGFR signaling at the distal convoluted tubule, which functions in the transepithelial magnesium transport, fails to maintain tubular integrity through EGFR.	Electrolyte disturbance (hypomagnesemia, hypophosphatemia, hypokalemia), diffuse proliferative glomerulonephritis, nephrotic syndrome, hypoalbuminemia	Nephrotic syndrome management through fluid and sodium restriction, oral or IV diuretics, and ACE inhibitors; magnesium wasting management by IV magnesium infusion and oral magnesium supplementation; discontinuation
mTOR inhibitors (Temsirolimus)	Inconclusive and multifactorial mechanism with possible iincreased glomerular permeability and injury, and suppression of tubular renal cell compensatory proliferation/survival/repair processes.	Glomerulopathy, AKI, proteinuria	Close monitoring of proteinuria and renal damage; early use of ACE inhibitors and ARBs with sirolimus; discontinuation
B-Raf inhibitors (Vemurafenib)	Damage to proximal tubules, inhibiting tubular secretion; reduction in GFR and creatinine clearance	Acute interstitial nephritis, acute tubular necrosis, AKI, Fanconi’s syndrome, hypertension	Routine monitoring of serum creatinine and electrolytes; discontinuation
Anti-angiogenesis (VEGF and VEGFR inhibitors) (Bevacizumab, Sorafenib, Sunitinib)	Anti-VEGF antibodies inhibit endothelial cell proliferation and blood vessel formation, resulting in loss of the filtration barrier; nitric oxide pathway inhibition and oxidative stress induce endothelial dysfunction and capillary rarefaction.	Nephrotic syndrome with high-grade proteinuria, AKI, TMA, and hypertension	Hypertension management through ACE inhibitors, ARBs, and discontinuation
Immune Checkpoint Inhibitors (Ipilimumab, Pembrolizumab, Nivolumab)	Enhanced T cell response with migration of activated T cells into the kidney; immune responses leading to inflammatory cell infiltrates; podocyte effacement	Acute tubulointerstitial nephritis, immune complex glomerulonephritis, TMA, AKI with possible granulomas	Corticosteroids; discontinuation
CAR-T therapy	CAR-T cell expansion and stimulation of the immune cell-secreting cytokines; fever, hypotension, renal failure	CRS; AKI	Tocilizumab
Cytokine therapy (IL-2)	Activation of TNF-alpha and other cytokines to induce capillary leak syndrome and renal hypoperfusion	Pre-renal azotemia; AKI	Fluid bolus; intermediate-dose dopamine; discontinuation

**Table 4:** Patient-related risk factors and agent-specific preventive strategies for drug-induced nephrotoxicity.

Patient Risk Factors and Agent-Specific Prevention Measured			References
Medication	Risk Factors	Prevention Strategies	
The pharmacological manipulation of intraglomerular hemodynamics (ACE inhibitors, ARBs, NSAID)	Acute renal injury can result from several identifiable factors. Concurrent administration of ACE inhibitors, ARBs, NSAIDs, cyclosporine, or tacrolimus, together with underlying renal insufficiency, decreased intravascular volume, advanced age (particularly in those aged 60 and beyond), and specific medications.	The prolonged use of acetaminophen, Aspirin, sulindac, and nabumetone is advised due to their pain-relieving effects. Prior consideration of replacing lost volume before the commencement of pharmaceutical management is recommended, especially when the drug is to be used long-term. Careful monitoring of renal function and vital signs is required when the drug is used in patients at high risk.	[22,35]
Cyclosporine, Tacrolimus	Furthermore, it is crucial to take into account the possible hazards linked to high dosage and the simultaneous use of other nephrotoxic medications or inhibitors of cyclosporine or tacrolimus metabolism.	Vigilant observation and analysis of medication concentrations in serum, together with monitoring renal function, is highly advised. Administering the minimal effective dosage is advised.	[128,180]
Drugs associated with tubular cell toxicity (Aminoglycosides)	Multiple variables can contribute to the onset of renal insufficiency, such as extended treatment (>10 days), aminoglycoside levels >2 µg/mL, the existence of liver disease, and hypoalbuminemia.	Administer drugs at long dose intervals.The drug should be administered at the time of peak activity during the day. Attempt to minimize therapy duration and add monitoring of serum drug concentrations and renal function every 2 to 3 weeks. To keep trough concentrations below 1 mcg/mL.	[37,86,138]
Amphotericin B (Fungizone; brand not available in the United States)	Extended treatment durations, frequent administration of high doses, formulations rich in deoxycholate rather than lipids, and established renal failure.	Prior to and following administration, hydration with salt water. Administering it to the patient as a 24 h infusion may be the optimal approach. Formulate in liposomes. Reduce the duration of the therapy.	[81,194]
Contrast dye	Renal impairment, advanced age, diabetes, congestive heart failure, volume depletion, or experience of multiple exposures.	Administer the minimum effective dosage of low-osmolar contrast and strive to avoid performing multiple procedures within a 24–48 h timeframe. Participants will be administered a 0.9% saline or a sodium bicarbonate infusion (154 mEq/L) before and after the treatment. Abstain from using any NSAIDs or diuretics for a minimum of 24 h before and after the procedure. Assess the patient’s renal function within 24 to 48 h following the surgery. Incorporate acetylcysteine into the preoperative considerations.	[125]
Medications that have been linked to chronic interstitial nephritis (Acetaminophen, aspirin, NSAIDs)	Patients satisfying the listed criteria: A medical history of persistent pain; age above 60 years; female gender; sustained use of analgesic medication over 1 g per day for a duration exceeding two years.	Avoid prolonged use of analgesics, particularly if you are utilizing multiple agents. It is recommended that patients experiencing persistent discomfort be treated with alternative pharmaceuticals.	[20]
Lithium	An increase in drug levels	Medication levels should always be maintained within the approved therapeutic range—Abstain from volume depletion.	
Crystal nephropathy and the drugs that cause it (Acyclovir, methotrexate, sulfa- antibiotics, triamterene)	Volume deficiency, underlying renal impairment, excessive dosage, and given by the intravenous route.	It is advisable to either cease or reduce the suggested dosage. Maintaining adequate hydration is of utmost importance. Facilitate enhanced urine flow. The medicine should be administered orally.	[35,37]

## Data Availability

No new data were created or analyzed in this study. Data sharing is not applicable to this article.

## Abbreviations

ARF	Acute renal failure
AKI	Acute kidney injury
ESRD	End-stage renal disease
CKD	chronic kidney disease
NGAL	Neutrophil gelatinase-associated lipocalin
KIM-1	Kidney injury molecule-1
NAG	N-acetyl-glucosaminidase
B2M	Beta-2 microglobulin
TFF3	Trefoil factor 3
TMA	Thrombotic microangiopathy
GFR	Glomerular filtration rate
NSAIDs	Nonsteroidal anti-inflammatory drugs
ACEIs	Angiotensin-converting enzyme inhibitors
ARBs	Angiotensin receptor blockers
GBHP	Glomerular blood hydrostatic pressure
AMGs	Aminoglycosides
ATP	Adenosine triphosphate
ATN	Acute tubular necrosis
TNF-α	Tumor necrosis factor-alpha
MAPK	Mitogen-Activated Protein Kinase
ROS	Reactive oxygen species
GSH	Glutathione
AMG	Aminoglycoside
PAF	Platelet-activating factor
AIDS	Acquired immunodeficiency syndrome
DNA	Deoxyribonucleic acid
HBV	Hepatitis B virus
HIV	Human immunodeficiency virus
OAT1	Organic anion transporter 1
OTC	Over-the-counter
COX-1	Ccyclooxygenase-1
BUN	Blood urea nitrogen
ROC	Receiver operating characteristic
PKD	Polycystic kidney disease
FSGS	Focal segmental glomerulosclerosis
IL-18	Interleukin-18
CIN	Contrast-induced nephropathy
KDIGO	Kidney disease: Improving Global Outcomes
3D	Three-dimensional
2D	Two-dimensional
OCT2	Organic cation transporter 2
